# DNA repair- and nucleotide metabolism-related genes exhibit differential CHG methylation patterns in natural and synthetic polyploids (*Brassica napus* L.)

**DOI:** 10.1038/s41438-021-00576-1

**Published:** 2021-07-01

**Authors:** Liqin Yin, Zhendong Zhu, Liangjun Huang, Xuan Luo, Yun Li, Chaowen Xiao, Jin Yang, Jisheng Wang, Qiong Zou, Lanrong Tao, Zeming Kang, Rong Tang, Maolin Wang, Shaohong Fu

**Affiliations:** 1 Institute of Crop Research, Chengdu Academy of Agricultural and Forestry Sciences, 200 Nongke Road, Chengdu, China; 2 College of Life Sciences, Sichuan University, 29 Wangjiang Road, Chengdu, China; 3 Agricultural College, Sichuan Agricultural University, 211 Huimin Road, Chengdu, China

**Keywords:** Evolution, Epigenomics, Genomic instability, Gene expression, Plant evolution

## Abstract

Polyploidization plays a crucial role in the evolution of angiosperm species. Almost all newly formed polyploids encounter genetic or epigenetic instabilities. However, the molecular mechanisms contributing to genomic instability in synthetic polyploids have not been clearly elucidated. Here, we performed a comprehensive transcriptomic and methylomic analysis of natural and synthetic polyploid rapeseeds (*Brassica napus*). Our results showed that the CHG methylation levels of synthetic rapeseed in different genomic contexts (genes, transposon regions, and repeat regions) were significantly lower than those of natural rapeseed. The total number and length of CHG-DMRs between natural and synthetic polyploids were much greater than those of CG-DMRs and CHH-DMRs, and the genes overlapping with these CHG-DMRs were significantly enriched in DNA damage repair and nucleotide metabolism pathways. These results indicated that CHG methylation may be more sensitive than CG and CHH methylation in regulating the stability of the polyploid genome of *B. napus*. In addition, many genes involved in DNA damage repair, nucleotide metabolism, and cell cycle control were significantly differentially expressed between natural and synthetic rapeseeds. Our results highlight that the genes related to DNA repair and nucleotide metabolism display differential CHG methylation patterns between natural and synthetic polyploids and reveal the potential connection between the genomic instability of polyploid plants with DNA methylation defects and dysregulation of the DNA repair system. In addition, it was found that the maintenance of CHG methylation in *B. napus* might be partially regulated by *MET1*. Our study provides novel insights into the establishment and evolution of polyploid plants and offers a potential idea for improving the genomic stability of newly formed *Brassica* polyploids.

## Introduction

Polyploidization is an important driving force in the evolution of angiosperms^1–3^. Most seed plants have undergone multiple cycles of genome duplication in their evolutionary history^2–5^. However, newly formed polyploids always face difficulties such as meiotic abnormalities and genomic and epigenetic instabilities during the early stages of evolution^6,7^, whereas established natural polyploids possess stable genomic properties. The genetic and epigenetic basis for the establishment and success of polyploid species is not fully understood. Analysis of genomic data indicated that angiosperm paleopolyploidizations were concentrated around the Cretaceous-Paleogene extinction event^8^, and the occurrence of polyploid plants was not random but predominantly depended on the environmental and ecological conditions^5,9^. Compared with genetic regulation, epigenetic modification is more susceptible to internal and external environmental cues and may play an important role in the establishment and survival of polyploid plants.

DNA methylation is a major epigenetic modification, and it often involves three kinds of sequence contexts in plant genomes, CG, CHG, and CHH (H = A, T, or C), each of which is maintained by several different but functionally overlapping enzymes. Methylation in CG contexts is maintained by MET1 (DNA methyltransferase 1), CHG by CMT3 (chromomethylase 3), and CHH by RdDM (RNA-directed DNA methylation) and CMT2 (chromomethylase 2)^10^. DNA methylation levels are dynamically regulated by DNA methylation and demethylation reactions. DNA methylation has an important role in developmental regulation, stress responses, and genome stability^10,11^. Disruption of DNA methylation can lead to abnormal development in plants and mammals^10,11^. However, in allopolyploid plants with complex genomes, the relationship between DNA methylation and genomic instability and the mechanism by which DNA methylation regulates genomic stability remain largely unclear.

*Brassica napus* (AACC, 2*n* = 38) is a recent allotetraploid species that originated ~7500 years ago from hybridization between *Brassica rapa* (AA, 2*n* = 20) and *Brassica oleracea* (CC, 2*n* = 18)^12^. Since the origin of the angiosperms, *B. napus* has undergone a predicted 72× polyploidization^12^. *B. napus* can be readily synthesized by interspecific hybridization and tissue culture and are considered a useful system for the investigation of polyploid genome evolution^12,13^. Accumulating evidence confirms the widespread genomic instability of newly synthesized *B. napus*^14–20^. Previous studies have demonstrated that synthetic *B. napus* has extensive changes in DNA methylation relative to its diploid parents (*B. rapa* and *B. oleracea*), which may contribute to the stability of its genome^15–17,21^. Changes in DNA methylation occurred as early as the first generation in synthetic *B. napus*, and most of these changes remained fixed in their fifth self-pollinated generation^15,16^. These studies have provided valuable insights into the effects of polyploidization on DNA methylation. However, these results were based on the techniques of restriction fragment length polymorphism^15,16^ or methylation-sensitive amplification polymorphism^17,21^, which could not accurately reflect changes in DNA methylation at the genome-wide level. Furthermore, related studies have mainly focused on the changes in DNA methylation levels and sites between synthetic *B. napus* and its diploid parents, and the biological functions of DNA sequences that undergo methylation changes in synthetic *B. napus* have not been fully elucidated. The association between DNA methylation and genomic instability of polyploid *B. napus* has not yet been clarified.

Natural *B. napus* has an evolutionary history of hundreds or thousands of years and is quite different from synthetic *B. napus* in terms of genomic stability and cytosine methylation^14,19,22,23^. A direct comparison between natural *B. napus* and synthetic *B. napus* may help to decipher the epigenetic evolutionary mechanism of the *B. napus* genome. A comparative analysis of genome resequencing data of synthetic and natural *B. napus* has revealed that homeologous exchanges drive genetic diversification and genome size reduction after polyploidization^24^. Genes involved in chromosome mismatch repair and chromosome stability are subject to deletions in natural *B. napus*^24^. However, the differences in genome-wide DNA methylation between synthetic and natural *B. napus* have received little attention.

Here, we investigated the differences in DNA methylation patterns between natural and synthetic polyploid rapeseeds by genome-wide bisulfite sequencing (WGBS). WGBS has been an important technique for detecting DNA methylation at a single base resolution level^25,26^ and has been used for methylation analysis in many plants, such as rice^27^, maize^28^, soybean^29^, tomato^30^, and apple^31^. Anthers are important reproductive organs of flowering plants and carry the genetic information to be passed on to the next generation. The DNA methylation patterns of anthers can be faithfully inherited during sexual development and across generations^32–35^. It is known that meiosis is the fundamental process of anther formation^36^, and the anthers of synthetic polyploids have a relatively high level of genomic instability. Therefore, anthers can be used to explore the potential mechanisms affecting the stability of polyploid genomes. However, most of the relevant studies on synthetic *Brassica* polyploids have focused on the vegetative growth stage where the somatic genomes are relatively stable^15–17,21^, whereas few studies have focused on the reproductive stage where the genomes are more unstable. In this study, we chose anthers instead of vegetable organs as the research materials. We wish to expand our understanding of the links between DNA methylation and genomic instability in synthetic polyploid *B. napus* and explore potential methods to improve the stability of the synthetic *B. napus* genome.

## Results

### Expression levels of DNA methylase and demethylase genes

**Fig. 1:**
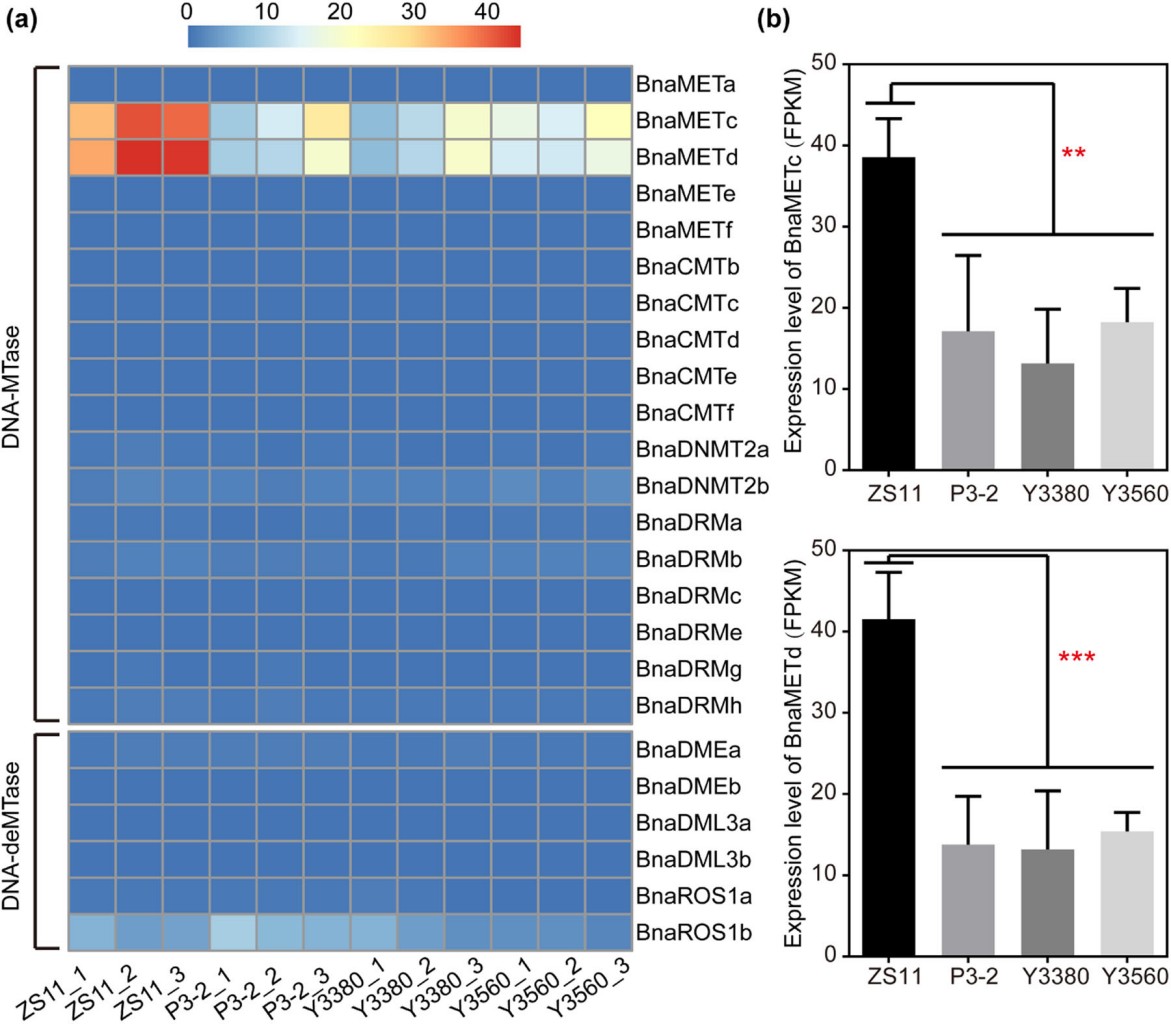
Expression patterns of genes coding DNA methylases (DNA-MTases) and DNA demethylases (DNA-deMTases) in mature anthers of *B. napus*. **a** Heat map of the expression levels (FPKM) of DNA-MTase and DNA-deMTase family genes. **b** Expression levels of two *MET1* genes. Statistical significance was analyzed by ANOVA. ***P < 0.001; **P < 0.01

DNA methylases and demethylases play important roles in the dynamic regulation of DNA methylation status in plants. To explore the expression differences in the potential DNA methylase and demethylase genes between natural and synthetic rapeseeds, we performed transcriptome analysis by RNA-seq to profile the gene expression levels (statistics of RNA-seq data can be found in Table S1). In total, 18 DNA methylase genes (5 *BnaMET1*, 5 *BnaCMT*, 2 *BnaDNMT2*, and 6 *BnaDRM*) and 6 DNA demethylase genes (2 *BnaDME*, 2 *BnaDML3*, and 2 *BnaROS1*) were identified (Table S2, Fig. 1a). Among them, 22 genes had very low expression levels and were not differentially expressed between natural and synthetic rapeseeds (Table S2, Fig. 1a). The *CMT* genes that help maintain cytosine methylation at CHG sites showed extremely low expression levels in all samples (Table S2). The expression levels of two *BnaMET1* genes (BnaC08g34700D and BnaA09g42250D) in synthetic rapeseeds were significantly lower than those in natural rapeseeds (Table S2, Fig. 1b). It was also found that the expression levels of other genes involved in the establishment, maintenance, and removal of DNA methylation were relatively low, and there were no obvious differences between natural *B. napus* and the synthetic rapeseeds (Table S2, Fig. S1).

### DNA methylomes of natural and synthetic polyploids

**Fig. 2:**
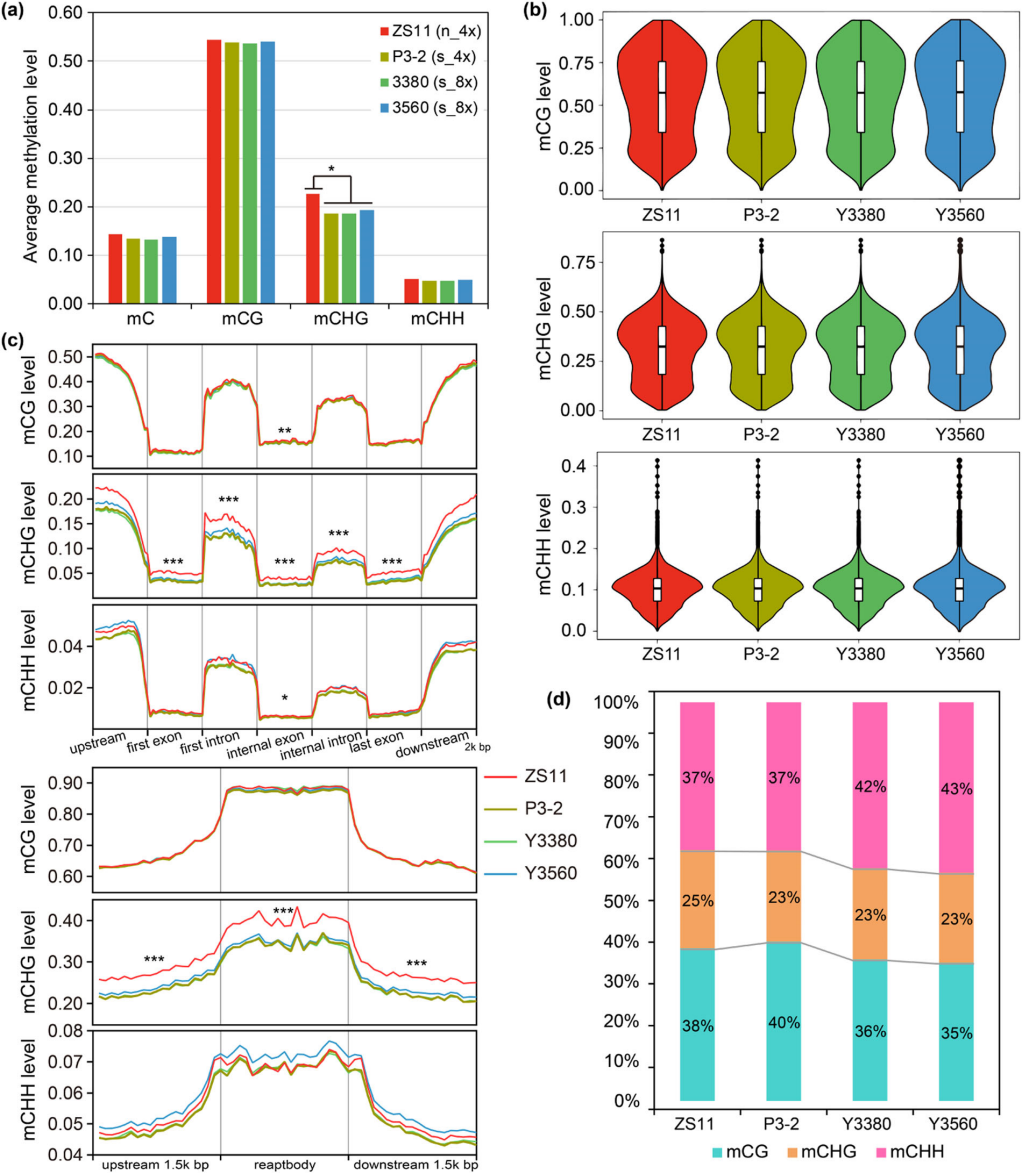
Landscape of DNA methylation in natural *B. napus* and synthetic *B. napus* polyploids. **a** Average methylation levels of total all C, CG, CHG, and CHH across the whole genome. *P < 0.05, by ANOVA. **b** Violin plot for the distributions of mCG levels, mCHG levels, and mCHH levels of the uniquely mapped reads. These methylcytosines were determined by using a binomial test, and the methylation levels were calculated according to Schultz et al.^96^. The ordinate represents the methylation level, and the width of each violin represents the density of points under the methylation level. Significant differences (*P* < 0.01) were detected between all samples by the Wilcoxon rank-sum test. **c** Average distribution of DNA methylation levels in gene regions and repeat regions. The methylation levels were compared between natural *B. napus* (ZS11) and synthetic *B. napus* (P3-2, Y3380, and Y3560) by ANOVA. ***P < 0.001; **P < 0.01; and *P < 0.05. **d** Proportions of mCs in each context (CG, CHG, and CHH) out of the total mCs

To uncover the differences in DNA methylation between natural and synthetic *B. napus* and explore the underlying mechanisms leading to chromosomal instability in synthetic polyploids, we performed WGBS analysis on natural (ZS11) and synthetic rapeseeds (P3-2, Y3380, and Y3560). By WGBS, 129.44 million and 103.36 million clean reads were generated from ZS11 and P3-2, respectively, with sequencing depths of ≥23×; 207.52 million and 204.72 million clean reads were filtered from Y3380 and Y3560, respectively, with sequencing depths of 42× (Table S3, Fig. S2). The natural *B. napus* ZS11 presented methylation of 14.24%, 54.56%, 22.55%, and 5.06% of the total sequenced C, CG, CHG, and CHH sites, respectively, reflecting average methylation levels across the whole genome (Table S4, Fig. 2a). In synthetic rapeseeds, the overall methylation levels of mC, mCG, mCHG, and mCHH were 13.38–13.72%, 53.74–54.08%, 18.46–19.32%, and 4.75–4.99%, respectively (Table S4, Fig. 2a). Compared with ZS11, the genome-wide mCHG levels of synthetic rapeseeds were significantly lower (Fig. 2a).

After removing duplicate reads, 53.29–107.01 million clean reads were uniquely mapped to the *B. napus* reference genome (Table S3). The methylation status of each cytosine with a sequencing depth ≥4 was determined by using a binomial test. The distributions of mCG levels, mCHG levels, and mCHH levels of the uniquely mapped reads showed that most mCG sites were highly methylated, whereas the majority of mCHG and mCHH sites were slightly methylated, especially mCHH sites (Fig. 2b). The four samples had significant differences in the distributions of cytosine methylation levels (*P* < 0.01, Wilcoxon rank-sum test) (Fig. 2b). The distributions of methylated cytosine sites on the chromosomes indicated that the C_n_ subgenomes of both natural and synthetic *B. napus* polyploids were more methylated than the A_n_ subgenomes (Figs. S3–S5).

**Fig. 3:**
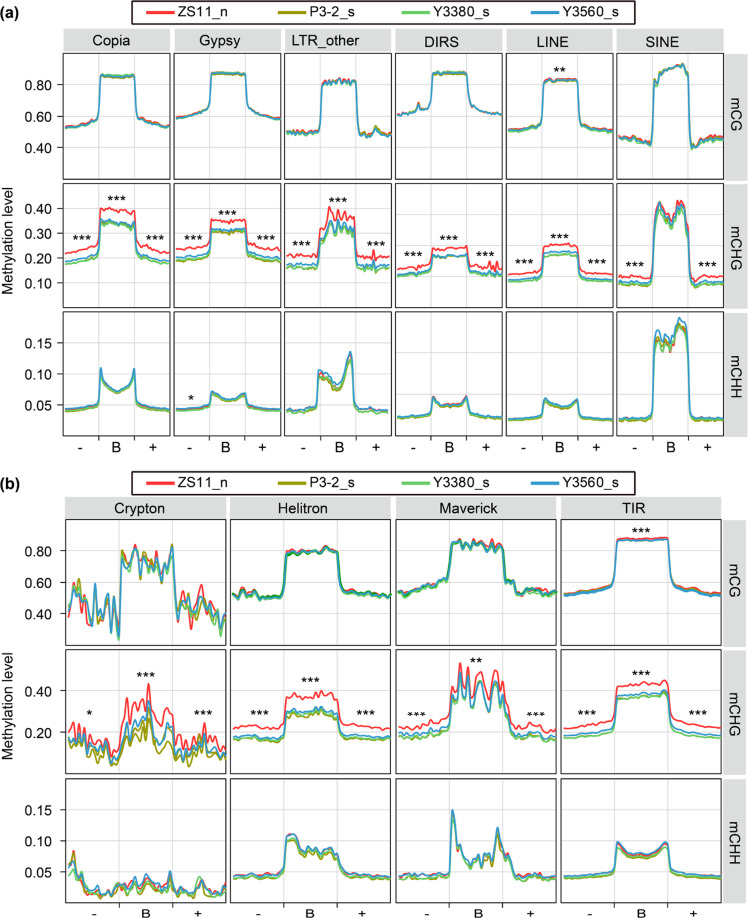
Average distribution of DNA methylation levels over different types of TEs. **a** Average distribution of DNA methylation levels on class I TEs. “−”, “B”, and “+” indicate upstream, body, and downstream regions of TEs, respectively. The upstream and downstream flanking regions are the same lengths as the TE body regions. **b** Average distribution of DNA methylation levels on class II TEs. The methylation levels were compared between natural *B. napus* (ZS11) and synthetic rapeseeds (P3-2, Y3380, and Y3560) by ANOVA. ***P < 0.001; **P < 0.01; and *P < 0.05

To understand the differences in the DNA methylation patterns of different genomic regions between natural and synthetic rapeseeds, we analyzed the methylation profiles of gene regions, repetitive sequences, and transposable elements (TEs). Extremely significant differences (*P* < 0.001, analysis of variance; ANOVA) were found in the mCHG levels of gene body regions and repetitive sequences (including upstream and downstream regions) between natural and synthetic rapeseeds (Fig. 2c). There were no significant differences (ANOVA) in the levels of mCG and mCHH in gene regions (except for the internal exons) and repetitive sequences between natural and synthetic rapeseeds (Fig. 2c). In addition to the decrease in mCHG levels, the proportions of mCHG sites in the total mC sites of synthetic rapeseeds were also slightly reduced (Fig. 2d). The detailed TE types were identified in the *B. napus* genome, and it was found that the average mCG levels of various types of TEs (*Copia*, *Gypsy*, *LTR-other*, *DIRS*, *LINE*, *SINE*, *Crypton*, *Helitron*, *Maverick*, and *TIR*) in the four rapeseeds were similar, whereas the mCHG levels of TEs in natural *B. napus* were obviously higher (Fig. S6). The most noticeable difference in the methylation patterns of TEs between natural and synthetic rapeseeds was found in the CHG context (Fig. 3). The mCHG levels in all types of TEs (including TE body, upstream and downstream regions, but not *SINE* bodies) in synthetic rapeseeds were significantly (*P* < 0.001, ANOVA) lower than those in natural *B. napus* (Fig. 3). Therefore, there were significant differences in the level and pattern of mCHG between natural and synthetic *B. napus* polyploids.

### DMRs between natural and synthetic rapeseeds

**Fig. 4:**
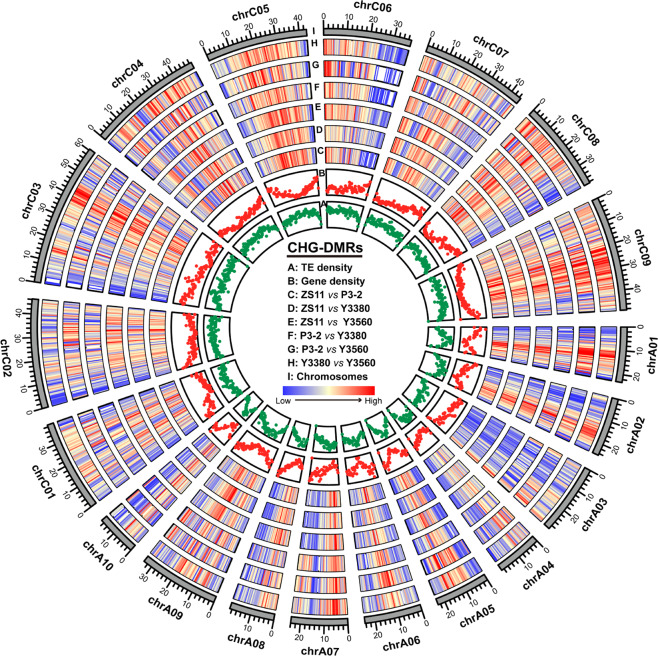
Genome-wide distribution of CHG-DMRs. Track order from inside to outside: the density of TEs; density of genes; density of CHG-DMRs from ZS11 vs P3-2; density of CHG-DMRs from ZS11 vs Y3380; density of CHG-DMRs from ZS11 vs Y3560; density of CHG-DMRs from P3-2 vs Y3380; density of CHG-DMRs from P3-2 vs Y3560; density of CHG-DMRs from Y3380 vs Y3560; and chromosomes. The circos plot was generated by TBtools^102^

**Fig. 5:**
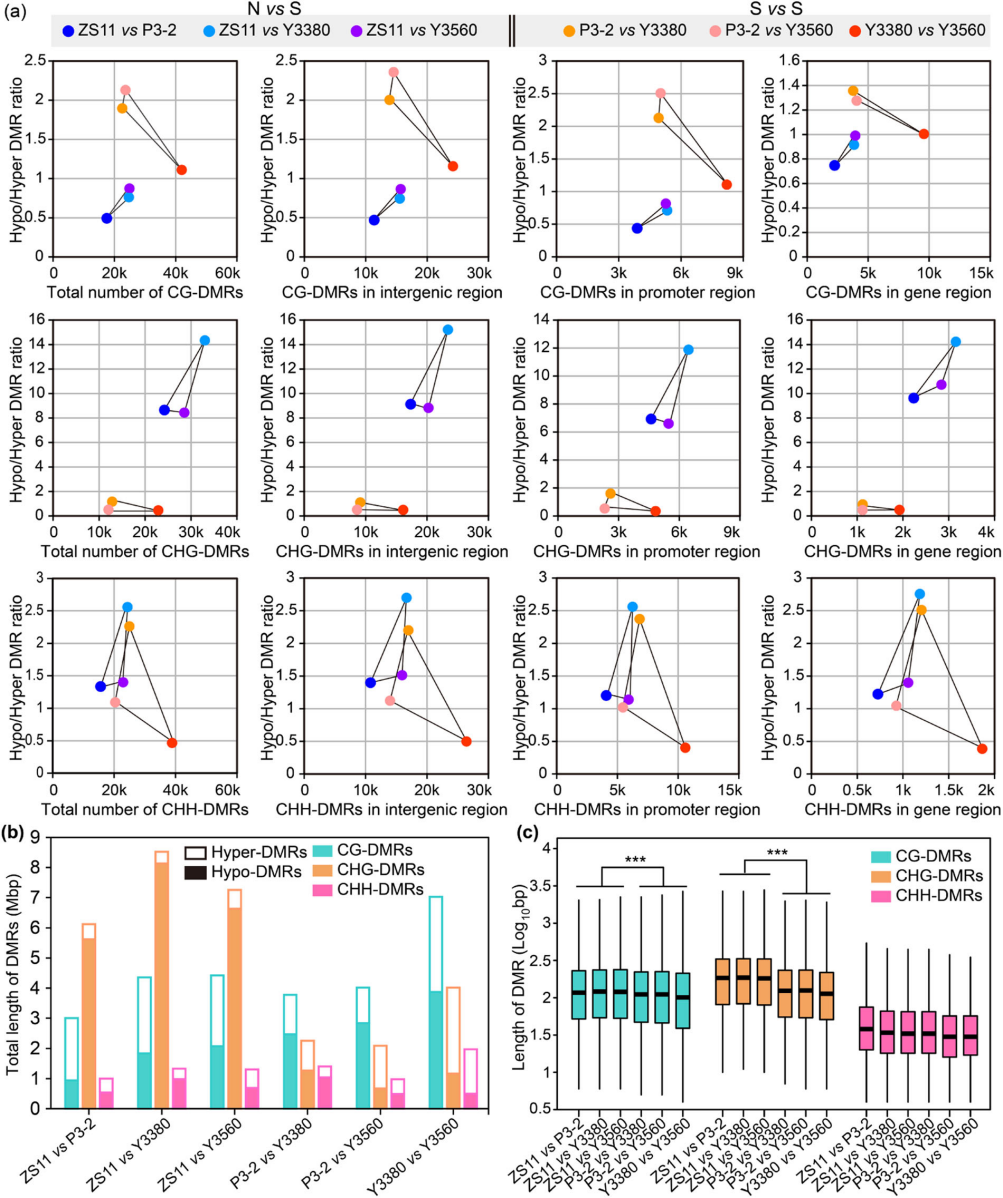
Characterization of DMRs between samples. **a** Scatter plots of DMRs, where each dot represents one comparison (ZS11 vs P3-2, ZS11 vs Y3380, ZS11 vs Y3560, P3-2 vs Y3380, P3-2 vs Y3560, and Y3380 vs Y3560). The abscissa represents the number of DMRs, and the ordinate indicates the Hypo-DMR/Hyper-DMR ratio. N vs S represents the comparisons between natural and synthetic rapeseeds, and S vs S represents the comparisons between synthetic rapeseeds. **b** The summed length of different types of DMRs for each comparison. The total lengths of CHG-DMRs from the N vs S comparisons were significantly longer than those from the S vs S comparisons (*P* = 0.0086, *t* test). **c** Box plot of the length of different types of DMRs. Statistical significance was analyzed by both ANOVA and the Wilcoxon rank-sum test. ***P < 0.001

To examine the DNA methylation variations between natural and synthetic rapeseeds, the differentially methylated regions (DMRs) between samples were identified. The genome-wide distributions of CG-DMRs, CHG-DMRs, and CHH-DMRs are shown (Fig. 4, S7–S8). Most of the CG-DMRs, CHG-DMRs, and CHH-DMRs were distributed in the distal intergenic regions, followed by promoter regions, and with the least found in gene body regions (Fig. S9). In the comparisons between natural and synthetic rapeseeds (N vs S), 17,515–24,910 CG-DMRs, 24,201–33,023 CHG-DMRs, and 15,620–24,392 CHH-DMRs were identified (Fig. 5a, Table S5). The average number of CHG-DMRs (28,595) from the N vs S comparisons was greater than that of CG-DMRs (22,380) and CHH-DMRs (20,983) (Table S5). In addition, the summed length of CHG-DMRs from each N vs S comparison was significantly longer than that of CG-DMRs (*P* = 0.01, *t* test) and CHH-DMRs (*P* = 0.009, *t* test) (Fig. 5b). Moreover, the average length of CHG-DMRs from N vs S comparisons was also significantly longer than that of CG-DMRs and CHH-DMRs (Fig. 5c). The DMRs between natural and synthetic rapeseeds were mainly concentrated in the CHG context. In contrast, the average amount of CHG-DMRs (15,921) in the comparisons between synthetic *B. napus* (S vs S) was much lower than that of CG-DMRs (29,376) and CHH-DMRs (28,092) (Fig. 5a, Table S5), and the summed length of CHG-DMRs was shorter (*P* = 0.041, *t* test) than that of CG-DMRs (Fig. 5b). The amount, total length, and average length of CHG-DMRs from the N vs S comparisons were all greater than those from the S *vs* S comparisons (Fig. 5, Table S5). Taken together, these results indicated that there were significant differences in DNA methylation between natural *B. napus* and synthetic rapeseeds, especially in the CHG sequence context.

It was also found that the total amount of hypomethylated CHG-DMRs was much greater than that of hypermethylated CHG-DMRs in the N vs S comparisons, the former being 8.5–14.3 times the latter, whereas the hypo/hyper CG-DMR ratio was only 0.5–0.9 and the hypo/hyper CHH-DMR ratio was 1.3–2.6 (Fig. 5a). The total length of hypo-CHG-DMRs was 10.7–21.2 times that of hyper-CHG-DMRs (Fig. 5b). Hypomethylation in the CHG context could be an important epigenetic feature of the genome of synthetic rapeseeds.

### DMGs between natural and synthetic rapeseeds

**Fig. 6:**
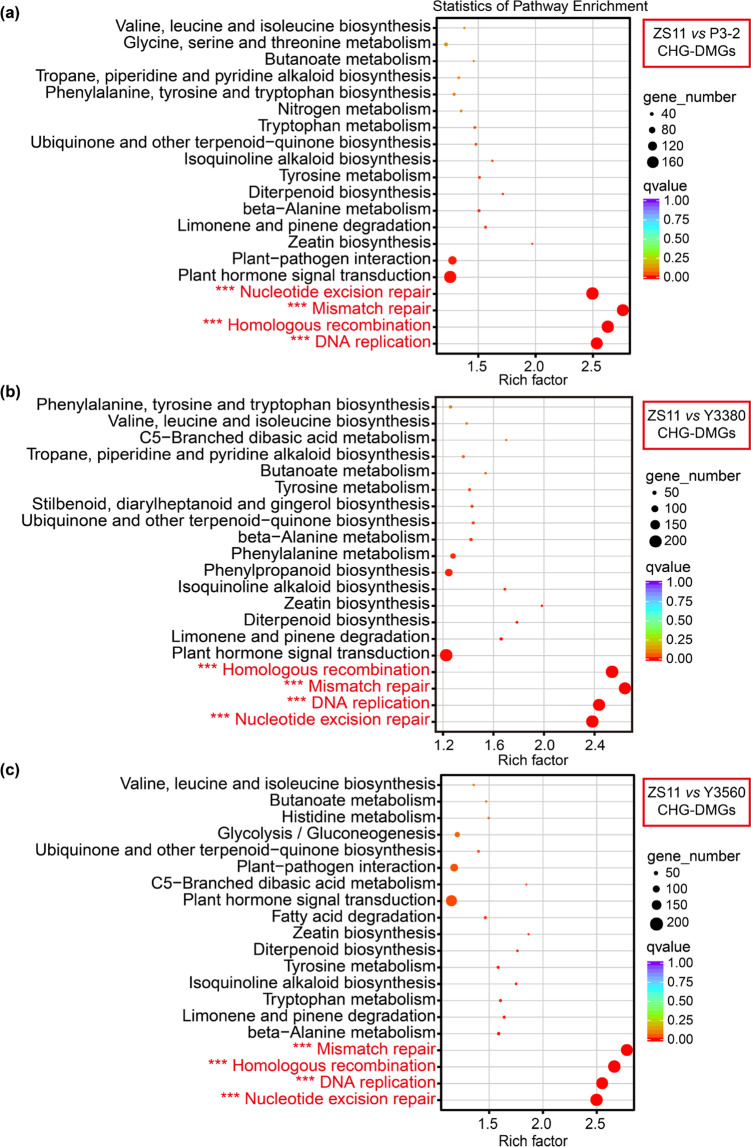
KEGG functional enrichment of CHG-DMGs between natural and synthetic rapeseeds. The top 20 enriched pathways are shown. **a** KEGG functional enrichment of CHG-DMGs from ZS11 vs P3-2. **b** KEGG functional enrichment of CHG-DMGs from ZS11 vs Y3380. **c** KEGG functional enrichment of CHG-DMGs from ZS11 vs Y3560. The rich factor is the ratio of the number of DMGs to the total number of genes in a certain pathway, which represents the degree of pathway enrichment. The asterisk indicates significant enrichment. ***Corrected *P* value < 0.001

**Fig. 7:**
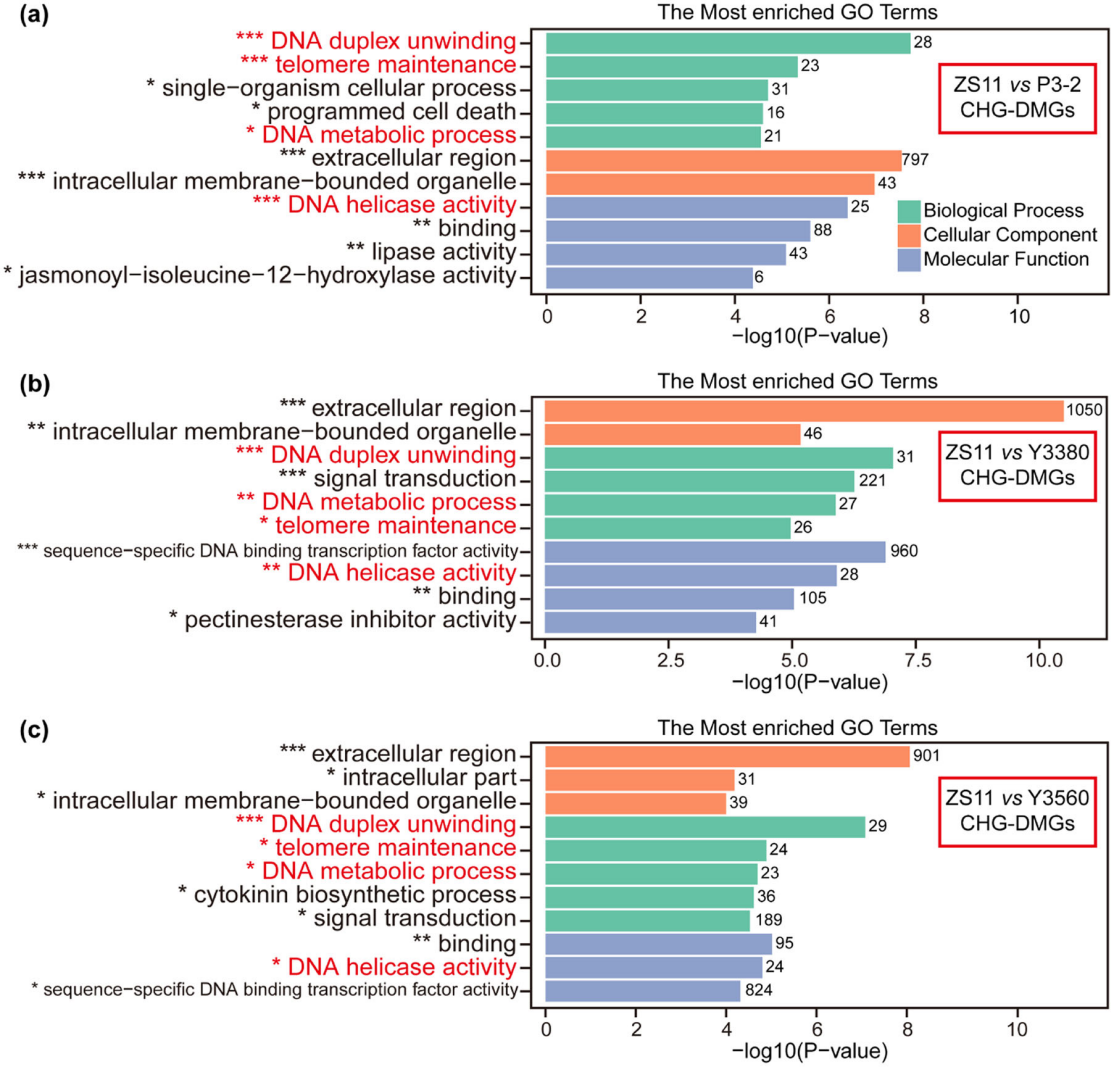
GO functional enrichment of CHG-DMGs between natural *B. napus* and synthetic *B. napus*. Only the significant GO terms (corrected *P* value <0.05) are listed. **a** GO functional enrichment of CHG-DMGs from ZS11 vs P3-2. **b** GO functional enrichment of CHG-DMGs from ZS11 vs Y3380. **c** GO functional enrichment of CHG-DMGs from ZS11 vs Y3560. ***, corrected *P* value < 0.001; **, corrected *P* value < 0.01; *, corrected *P* value < 0.05

To investigate the biological functions of genes overlapping with DMRs (differentially methylated genes (DMGs)), enrichment analysis was performed for KEGG (Kyoto Encyclopedia of Genes and Genomes) pathways and GO terms. The results showed that the CHG-DMGs between natural and synthetic rapeseeds were significantly (corrected *P* value < 0.001) enriched in the pathways “nucleotide excision repair”, “DNA replication”, “mismatch repair”, and “homologous recombination” (Fig. 6) and significantly enriched in the GO terms “DNA duplex unwinding”, “DNA metabolic process”, “telomere maintenance”, and “DNA helicase activity” (Fig. 7). These enriched genes are believed to play an important role in maintaining chromosomal stability. More than 90% of these enriched CHG-DMGs were hypomethylated in synthetic rapeseeds (Tables S6–S7). The common hypo-CHG-DMGs across the three N vs S comparisons were also enriched in these eight important pathways and GO terms (Fig. S10). In addition, many CHG-DMGs (N vs S) were involved in biological processes related to meiosis, mitosis, DNA, and nucleotide metabolism (Table S8).

Although the CG-DMGs and CHH-DMGs between natural and synthetic rapeseeds were significantly enriched in the above four pathways (Figs. S11–S13, Table S6), the number of CG-DMGs and CHH-DMGs enriched in these pathways was much less than that of CHG-DMGs (Table S6). Moreover, the CG-DMGs and CHH-DMGs from the N vs S comparisons were not significantly enriched in the above four GO terms (Figs. S11–S13, Table S6). These results indicated that the differences in DNA methylation between natural and synthetic rapeseeds, especially in CHG methylation, might be highly correlated with the ability to maintain genome stability.

To be more comprehensive, we analyzed the enrichment of DMGs among synthetic rapeseeds and found that they were significantly enriched in pathways such as “nucleotide excision repair”, “DNA replication”, “mismatch repair”, and “homologous recombination”, but there was almost no significant enrichment in the GO terms “DNA duplex unwinding”, “DNA metabolic process”, “telomere maintenance”, and “DNA helicase activity” (Table S6). The numbers of enriched DMGs and DMRs were relatively small (Table S7). These results suggested that the differences in DNA methylation between synthetic rapeseeds seem to be mainly concentrated in the CG and CHH contexts.

### Differentially expressed genes between natural and synthetic rapeseeds

**Fig. 8:**
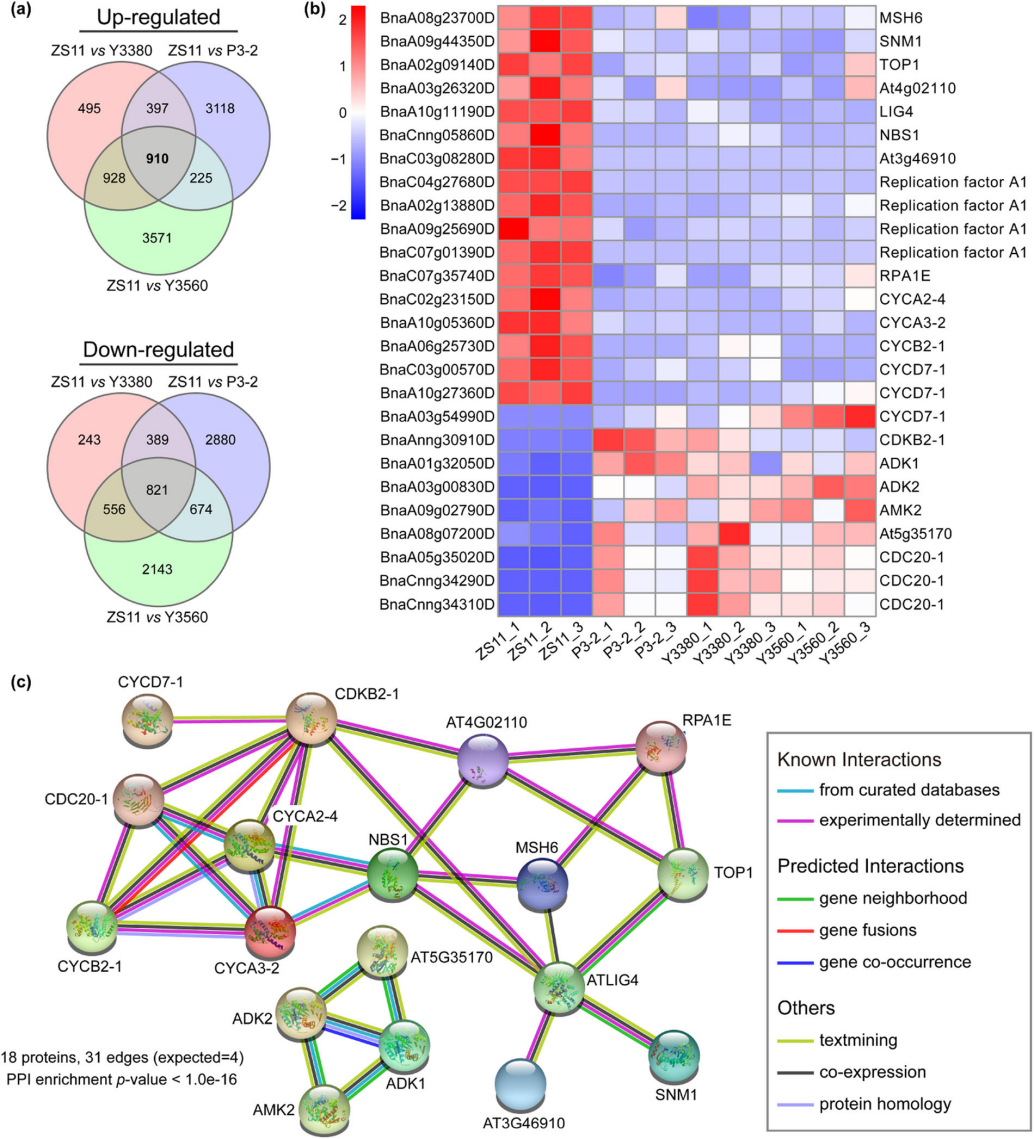
Differentially expressed genes between natural *B. napus* and synthetic *B. napus*. **a** Venn diagrams of DEGs (upregulated and downregulated, fold change >2, FDR < 0.01) from three comparisons of N vs S. **b** Heat map showing the expression patterns of DEGs related to the DNA damage repair system. **c** STRING analysis of the interaction networks of the DEGs related to the DNA damage repair system. All information for these genes can be found in Tables S9 and S10

To determine whether the genes involved in the maintenance of genome stability were differentially expressed in natural and synthetic rapeseeds, we analyzed the common differentially expressed genes (DEGs) of the three N vs S comparisons. A total of 1731 DEGs (910 upregulated and 821 downregulated) were detected between natural and synthetic rapeseeds (Fig. 8a, Table S9). Among the 1731 DEGs, ~272 DEGs could be related to DNA metabolism and chromosome stability (Table S9). The genes encoding DNA mismatch repair protein (MSH6), DNA crosslink repair protein (SNM1), DNA topoisomerase 1 (TOP1), DNA ligase IV (LIG4), Nijmegen breakage syndrome 1 protein (NBS1), and BRCT domain-containing protein were significantly downregulated in synthetic rapeseeds (Fig. 8b). These genes are directly involved in DNA repair and play a critical role in the cellular response to DNA damage and the maintenance of chromosome integrity (Table S10). The replication factor A genes required for DNA recombination, repair, and replication were downregulated in synthetic rapeseeds (Fig. 8b, Table S10). The cyclin (CYC), cyclin-dependent kinase (CDK), and cell division cycle (CDC) genes, which are involved in the regulation of the cell cycle and cell division, were differentially expressed between natural and synthetic rapeseeds (Fig. 8b, Table S10). In addition, the adenosine kinase (ADK) and adenylate kinase (AMK) genes that play an important role in nucleotide metabolism were significantly upregulated in synthetic rapeseeds (Fig. 8b, Table S10). The interaction networks of these key DEGs are shown in Fig. 8c. These results indicated that many genes involved in DNA repair and metabolism were differentially expressed between natural and synthetic rapeseeds.

In addition, to gain insight into the correlation between DNA methylation and gene expression, we compared the DMGs and DEGs between samples. The number of DMGs was much greater than that of DEGs, and there was little overlap between DMGs and DEGs (Fig. S14a). Changes in the DNA methylation levels of most genes did not directly affect the expression levels of genes, and most DEGs were not directly related to changes in DNA methylation levels (Fig. S14a). Despite this, there were still some genes related to DNA repair and metabolism that were both differentially methylated and differentially expressed in natural and synthetic rapeseeds (Table S10, Fig. S14b). The relationship between DMRs and DEGs was complicated.

## Discussion

Polyploidization has played a prominent role in the evolution of angiosperms^1–3,5^. However, the mechanisms for the successful establishment of polyploid species remain largely unclear, and the molecular mechanisms leading to genomic instability in synthetic polyploids have not been clearly elucidated^37,38^. We compared the differences in genome-wide DNA methylation between natural and synthetic rapeseeds and found that the most significant difference between them was reflected in the CHG context. The mCHG levels of most genomic regions (gene regions, transposon regions, and repetitive sequence regions) of the synthetic rapeseeds were significantly lower than those of natural *B. napus*. The total amount and summed length of CHG-DMRs between natural and synthetic rapeseeds were both much greater than those of CG-DMRs and CHH-DMRs. Keeping a proper state of DNA methylation is important for genome stability^39^. Recent studies have demonstrated that disruption of *Arabidopsis thaliana* non-CG methylation pathways increases pericentromeric crossovers and DNA double-strand breaks during meiosis, leading to chromosome missegregation and aneuploidy^40,41^. The abnormal patterns of DNA methylation are also a conspicuous feature of most human cancer cells that have unstable genomes^42–46^. Hypomethylation in the CHG context may be an epigenetic characteristic of the genome of synthetic rapeseed polyploids. The significant reduction in the CHG methylation level and the rearrangement of a large number of methylated cytosines in synthetic rapeseeds may severely disrupt the methylation balance of *B. napus* species and cause chromosomal instability in the newly formed polyploids.

Defects in DNA methylation help promote genetic lesions in human cancer^42^. However, there are few studies on epigenetic modifications that contribute to the DNA damage response and DNA repair in plants^47^, especially in polyploid plants. Our data indicated that the CHG-DMGs between natural and synthetic rapeseeds were significantly enriched in the pathways “DNA replication”, “mismatch repair”, “nucleotide excision repair”, and “homologous recombination” and were also significantly enriched in the biological processes of “DNA double helix unwinding”, “DNA metabolism”, “DNA unwinding activity”, and “chromosome telomere maintenance”. All these pathways and biological processes are highly linked to DNA damage repair and DNA metabolism^48,49^. In eukaryotes, DNA damage repair pathways help to maintain the integrity and stability of genetic and epigenetic information^50,51^. DNA damage is a common phenomenon in cell growth and metabolism, and cells have established a variety of DNA damage detection, identification, and repair systems to ensure the normal functions of organisms^50^. The inability to repair DNA damage efficiently may lead to genetic mutations and chromosomal instability^50^. Our results demonstrated that the expression levels of some important DNA repair genes (such as *MSH6*, *SNM1*, *TOP1*, *LIG4*, and *NBS1*) in synthetic rapeseeds were significantly lower than those in natural rapeseed. The mismatch repair protein MSH6 is involved in recognizing base mismatches and short insertion/deletion loops in most eukaryotes^52^. The disruption of DNA mismatch repair genes affects genomic stability and causes genome-wide methylation changes^53,54^; *MSH6* mutation in mammalian cells frequently induces several cancers^55–57^. SNM1 is a DNA structure-specific endonuclease and is required in vivo for repairing chromosomal breaks harboring closed hairpin ends^58^. TOP1, a main cellular factor that controls topological homeostasis, initiates ribonucleotide excision repair to remove ribonucleotides from genomic DNA^59^. DNA ligase IV (LIG4) mediates nonhomologous DNA end joining and is essential for DNA double-strand break repair^60–62^. NBS1 is essential for repairing DNA double-strand breaks through homologous recombination^63,64^. The lower expression levels of these DNA repair genes in synthetic rapeseeds would lead to unrepaired DNA damage, a high frequency of genetic mutations and chromosomal instability, which may be detrimental to the stability and survival of the species^65^ and may lead to chromosome loss and aneuploidy in the offspring of synthetic polyploids by self-pollination or hybridization. In addition, unrepaired DNA damage in gametes could be transmitted and accumulate through fertilization and genome replication^66–68^. The accumulation of DNA damage has profound effects on cell functions^69^. Collectively, the synthetic *B. napus* polyploid has defects in both the DNA methylation pattern and DNA repair system.

In addition, many genes involved in nucleotide metabolism, DNA replication, and cell cycle control were differentially expressed and differentially methylated between natural and synthetic rapeseeds. Synthetic rapeseeds may have defects in nucleotide metabolism and cell cycle regulation, which is consistent with a previous study showing that nucleotide pool imbalance may be a common response to polyploidization^70^. Precise regulation of nucleotide metabolism, DNA replication, the cell division cycle, and DNA damage repair is essential for the maintenance of genome stability and normal development^71,72^. Nucleotide metabolism imbalance could trigger genomic shock in newly formed autopolyploids^70^. Disorders of cell cycle progression present challenges for DNA damage repair^72^. Mutation of DNA repair genes can lead to DNA methylation changes^51,53^. Theoretically, factors affecting both DNA repair and DNA methylation may strongly impact the integrity or plasticity of the plant genome^51^. Previous studies indicated that DNA hypomethylation in human cells is generally characterized by DNA damage and is associated with increased chromosomal instability^73^. The causal link between DNA hypomethylation and chromosomal instability is one of the mechanisms leading to tumor formation^43,74^. In addition, DNA methylation and gene expression help regulate each other^10,75^. Therefore, we propose a putative model for the causes and processes contributing to genomic instability in newly formed polyploids. Genomic stress from neopolyploidization may affect chromatin structure, epigenetic modification, and gene expression, which may lead to chromosomal instability, epigenetic defects, and gene expression dysregulation. Chromosomal instability, epigenetic abnormalities, and gene expression dysregulation affect each other. These three factors cause DNA damage and disrupt the DNA repair system, nucleotide metabolism balance, and cell cycle progression, which in turn affect chromosomal stability, epigenetic regulation, and gene expression. Genomic instability can accumulate and be transmitted. Genetic and epigenetic damage drive each other to make the genetic environment more unstable, thus entering a cycle of genomic instability, which would eventually threaten the survival and establishment of the newly formed polyploids. If the internal and external environments are improved and the damage repair capacity is strong enough, the genome may gradually become stable, and relatively stable polyploid offspring could be obtained.

In addition, our results suggested that CHG methylation seems to be more sensitive than CG and CHH methylation in regulating the stability of the polyploid genome, which is consistent with the recent view that non-CG methylation is superior to CG methylation in genome regulation^76^. The functions of CHG methylation may be distinct from those of CG methylation. CHG methylation is stronger than CG methylation in silencing transposons^76^. Although both CG and non-CG DNA methylation inhibit centromeric meiotic doubled-strand breaks^40^, only non-CG methylation (especially, CHG methylation) inhibits crossovers in the pericentromeres^41^. Moreover, genic CHG methylation is associated with genome size, whereas the relative mCG levels among genes have remained stable since the divergence of ferns and angiosperms^77^. In addition, CHG methylation in plants may be more susceptible to external stress than CG methylation^78^. Gamma irradiation-induced *A. thaliana* DNA hypomethylation preferentially occurred at CHG or CHH sites instead of CG sites^79^. Heavy metal stress specifically induced CHG hypomethylation in rice^80^. The “genomic shock” in newly formed polyploids is actually an internal stress^39^. A previous study showed that the most obvious difference in TE methylation between natural diploid and synthetic autotetraploid rice occurred in the CHG context^81^. Collectively, variations in CHG methylation may be a common response of plants to internal and external stresses. Modulating CHG methylation may be a potential way to improve the genomic stability of synthetic polyploids.

Another interesting finding was that *CMT* homologous genes were almost not expressed in the mature anthers of *B. napus*, and the decrease in mCHG levels in synthetic rapeseeds may be caused by the lower expression levels of *MET1* genes. The expression levels of *CMT* genes in other organs of *B. napus* were also very low^21,82^, which is consistent with the view that the *CMT3* pathway was less effective in *Brassicaceae* genomes than in other plant species^83–85^. Although the mechanism for propagating CG methylation seems to be clearly defined, the mechanism for maintaining non-CG methylation in plants is less clear^86^. MET1 maintains CG cytosine methylation, and CMT3 maintains CHG methylation^10^. However, recent important results suggested the involvement of MET1 in the maintenance of methylation at CHG sites^86–88^. DNA methylation at the CCG site in *Physcomitrella patens* and *A. thaliana* depends on both *MET1* and *CMT3*, whereas CAG and CTG methylation only require *CMT3*^86–88^. The *met1* mutation in *A. thaliana* caused the specific loss of CCG methylation on the entire chromosome^87^. Our data showed that among almost all DNA methylase and DNA demethylase genes, only the *MET1* genes had significantly lower expression levels in synthetic rapeseeds, suggesting that *MET1* may be involved in the regulation of CHG methylation in *B. napus*. Interestingly, the lower expression levels of *MET1* did not significantly reduce the CG methylation level of synthetic rapeseeds, indicating that *MET1* could preferentially maintain CG methylation and then CHG methylation, which is consistent with the finding in *Arabidopsis* that maintenance of CHG methylation is not as efficient as that of CG methylation^84^. In *Arabidopsis* that have lost *CMT3* expression, genic CG methylation was preferentially maintained and inherited relative to genic CHG and CHH methylation^89^. The maintenance of CHG methylation in *Brassicaceae* species (*Eutrema salsugineum* and *Conringia planisiliqua*) that naturally lack *CMT3* may be more complicated and may be a result of RdDM^83^. Our results confirm the diversity and complexity of CHG methylation patterns in *Brassicaceae* plants and support the view that *MET1* is involved in the maintenance of CHG methylation.

In conclusion, our data highlight that the genes related to DNA repair and nucleotide metabolism display differential CHG methylation patterns between natural and synthetic polyploids. CHG methylation may play an important role in maintaining the stability of the polyploid *B. napus* genome. Our results have revealed the potential links between the genomic instability of polyploid plants with DNA methylation defects and the dysregulation of the DNA damage repair system. Genomic stress in synthetic rapeseeds could disrupt the DNA methylation balance of the species and disturb the regulation of DNA damage repair, nucleotide metabolism, and the cell cycle, which in turn would increase the instability and fragility of polyploid genomes. The maintenance mechanism of CHG methylation in *B. napus* may be partially regulated by *MET1*. Our study provides novel insights into the evolution of polyploid plants and offers potential ideas for improving the genomic stability of newly formed polyploids.

## Materials and methods

### Plant materials

Three synthetic *B. napus* lines were selected, including one synthetic tetraploid *B. napus* line (P3-2, AACC, 2*n* = 38) and two synthetic octoploid *B. napus* lines (Y3380 and Y3560, AAAACCCC, 2*n* = 76). P3-2 was derived from self-pollination of an artificially synthesized hexaploid *Brassica* (*B. napus* × *B. rapa*)^90^, and it has been self-pollinated for twelve generations. Its twelfth-generation self-progeny was used here. Y3380 and Y3560 were artificially synthesized by doubling the genome of allotetraploid *B. napus*^91^. They have undergone several generations of self-pollination, and their seventh-generation self-offspring were used here. Owing to the stable genetic characteristics, the natural *B. napus* variety ZS11 was selected as a control. The meiotic process of P3-2 was slightly abnormal, and a few inbred progenies with aneuploid chromosomes could be produced (Fig. S15). The synthetic octoploid *B. napus* faced the same problems of genome shock and instability as synthetic tetraploid *B. napus*, and their meiosis was more unstable owing to the increased number of chromosomes^90,91^. All these plant materials were grown under the same conditions in the experimental field in Wenjiang (E103.83, N30.70), Chengdu, China. Somatic chromosome counting, fluorescent in situ hybridization, and flow cytometry analysis were performed to screen P3-2 plants with a complete genome of *B. napus* and Y3380/Y3560 plants with twice the genome of *B. napus*^91^. During the flowering stage, the first five fully opened flowers were picked from five plants of each sample. The mature anthers were collected in duplicate (one for DNA extraction and the other for RNA extraction), frozen immediately in liquid nitrogen, and stored at −80 °C for the subsequent extraction of genomic DNA and total RNA.

### Cytological observation

Somatic chromosome counting and observation of meiotic behavior were carried out according to the procedures detailed in Li et al.^92^ and Zhou et al.^93^. Young ovaries were treated with 0.002 mol/L 8-hydroxyquinoline solutions for 3 h in the dark and then transferred to Carnoy’s fixative (ethanol: glacial acetic acid, 3:1, v/v) for more than 24 h. Young flower buds (2–3 mm long) were collected in Carnoy’s fixative (ethanol: glacial acetic acid, 3:1, v/v) in the morning and then fixed for 24 h at room temperature. Young ovaries and anthers were dissected out, incubated in 1 M hydrochloric acid solution at 60°C for 6–8 min, removed and placed on a glass slide, squeezed gently to release pollen mother cells into Carbol fuchsin solution, and then observed under an optical microscope.

### DNA extraction and bisulfite sequencing (BS-Seq)

Total genomic DNA (gDNA) was extracted from mature anthers according to a modified CTAB extraction protocol^94^. gDNA was fragmented by sonication to 100–500 bp with a Bioruptor sonicator (Diagenode, Belgium), followed by end-repair, adenylation, and methylated adapter ligation. Then, the DNA fragments were treated with bisulfite using the EZ DNA Methylation-Gold Kit (Zymo Research, USA). The bisulfite-treated DNA was amplified by PCR to construct a sequencing library. The concentration of the library was accurately quantified by a Qubit^®^ 2.0 Fluorometer (Life Technologies, USA) and StepOnePlus^TM^ Real-Time PCR system (Applied Biosystems, USA), and the insert size was detected on an Agilent Bioanalyzer 2100 Bioanalyzer (Agilent, USA). High-quality libraries were then sequenced on the Illumina HiSeq X Ten platform to generate 150-bp paired-end reads (BioMarker Technologies, Beijing, China).

### BS-Seq data analysis

The raw reads were filtered by removing adapter reads, reads with >10% Ns, and reads with >50% low-quality bases. Clean data were aligned to the *B. napus* reference genome (v4.1, http://www.genoscope.cns.fr/brassicanapus/data/) by Bismark software^95^ with default parameters. The bisulfite conversion rate, sequencing depth, and coverage were evaluated by using Bismark. Genome-wide average methylation levels in the CG, CHG, and CHH contexts were calculated as the proportion of methylated cytosine reads to the total cytosine (C) reads. Only uniquely mapped reads were retained for the detection and annotation of methylcytosines (mCs) and DMRs. The methylation status of each cytosine was determined by using a binomial test (R package) and false discovery rate (FDR < 0.05) correction, and only C sites with coverage by at least four reads were considered. The methylation level of a C site was calculated according to the formulas detailed in Schultz, et al.^96^. The relative proportion of mCs in the three sequence contexts was calculated as the percentage of mCG, mCHG, and mCHH in the total mC sites.

DMRs between samples were identified by MOABS software^97^, with read coverage ≥10, number of differentially methylated cytosines ≥3, difference in methylation levels ≥0.2 (0.3 for CG type) and Fisher’s exact test *p* value < 0.05. Genes overlapping with DMRs were characterized as DMGs. The DMGs were analyzed for GO (Gene Ontology) and KEGG enrichment. The GOseq R package was applied for GO enrichment analysis^98^, and KOBAS software was used to test the statistical enrichment of DMGs in KEGG pathways^99^. GO terms and KEGG pathways with corrected *p* values < 0.05 were considered significantly enriched by DMGs. TEs overlapping with DMRs were defined as differentially methylated TEs. The TEs were annotated by running RepeatMasker^100^ against the *B. napus* reference genome (v4.1), and the parameters were set according to Cheng, et al.^101^.

### RNA extraction and transcriptome sequencing

Total RNA was isolated from mature anthers using a Plant Total RNA Extraction Kit (Tiangen Biotech, China) and treated with RNase-free DNase I to remove genomic DNA contamination. RNA quality was determined using a NanoDrop 2000 spectrophotometer (Thermo Scientific, USA), and RNA integrity was assessed using an Agilent Bioanalyzer 2100 system (Agilent Technologies, USA). A total of 1 μg of high-quality RNA per sample was subjected to library construction using the NEBNext UltraTM RNA Library Prep Kit for Illumina (NEB, USA) following the manufacturer’s instructions. The library fragments were purified with the AMPure XP system (Beckman Coulter, USA) to preferentially select cDNA fragments that were ~240 bp in length. Clustering of the index-coded samples was performed on a cBot Cluster Generation System using TruSeq PE Cluster Kit v4-cBot-HS (Illumina). After cluster generation, the RNA library was sequenced on an Illumina HiSeq Xten platform, and 150 bp paired-end reads were generated. Three biological replicates were performed for each sample.

Clean data were obtained by removing reads containing adapters, reads containing poly-N sequences and low-quality reads from the raw data; the clean reads were then mapped to the *B. napus* reference genome (v4.1, http://www.genoscope.cns.fr/brassicanapus/data/) by TopHat2 and Bowtie2 tools. Only uniquely mapped reads were considered for further analysis. Gene expression levels were estimated by fragments per kilobase of transcript per million mapped reads. DEGs between samples were determined by using the DESeq2 program with an absolute value of the expression fold change ≥2 and FDR threshold ≤0.01. The function of DEGs was also analyzed using GO and KEGG tools.

## Supplementary information

Revised Supplemental Figures

Revised Supplemental Tables

## Data Availability

The sequencing data generated in this study have been deposited into the Sequence Read Archive (SRA) database in NCBI under accession number PRJNA627985.
