# Supplementary material for: DNA repair- and nucleotide metabolism-related genes exhibit differential CHG methylation patterns in natural and synthetic polyploids (Brassica napus L.)
Source: Hortic Res. 2021 Jul 1;8:142. doi: 10.1038/s41438-021-00576-1 (PMC8245426; doi:10.1038/s41438-021-00576-1)
Supplement: Supplementary file 1 — Revised Supplemental Figures [file 41438_2021_576_MOESM1_ESM.doc]

**DNA repair- and nucleotide metabolism-related genes exhibit differential CHG methylation patterns in natural and synthetic polyploids (*Brassica napus* L.)**

Liqin Yin^1,2*^, Zhendong Zhu^1^, Liangjun Huang^1,3^, Xuan Luo^1,3^, Yun Li^1^, Chaowen Xiao^2^, Jin Yang^1^, Jisheng Wang^1^, Qiong Zou^1^, Lanrong Tao^1^, Zeming Kang^1^, Rong Tang^1^, Maolin Wang^2*^, Shaohong Fu^1*^

**Supplemental Figures**


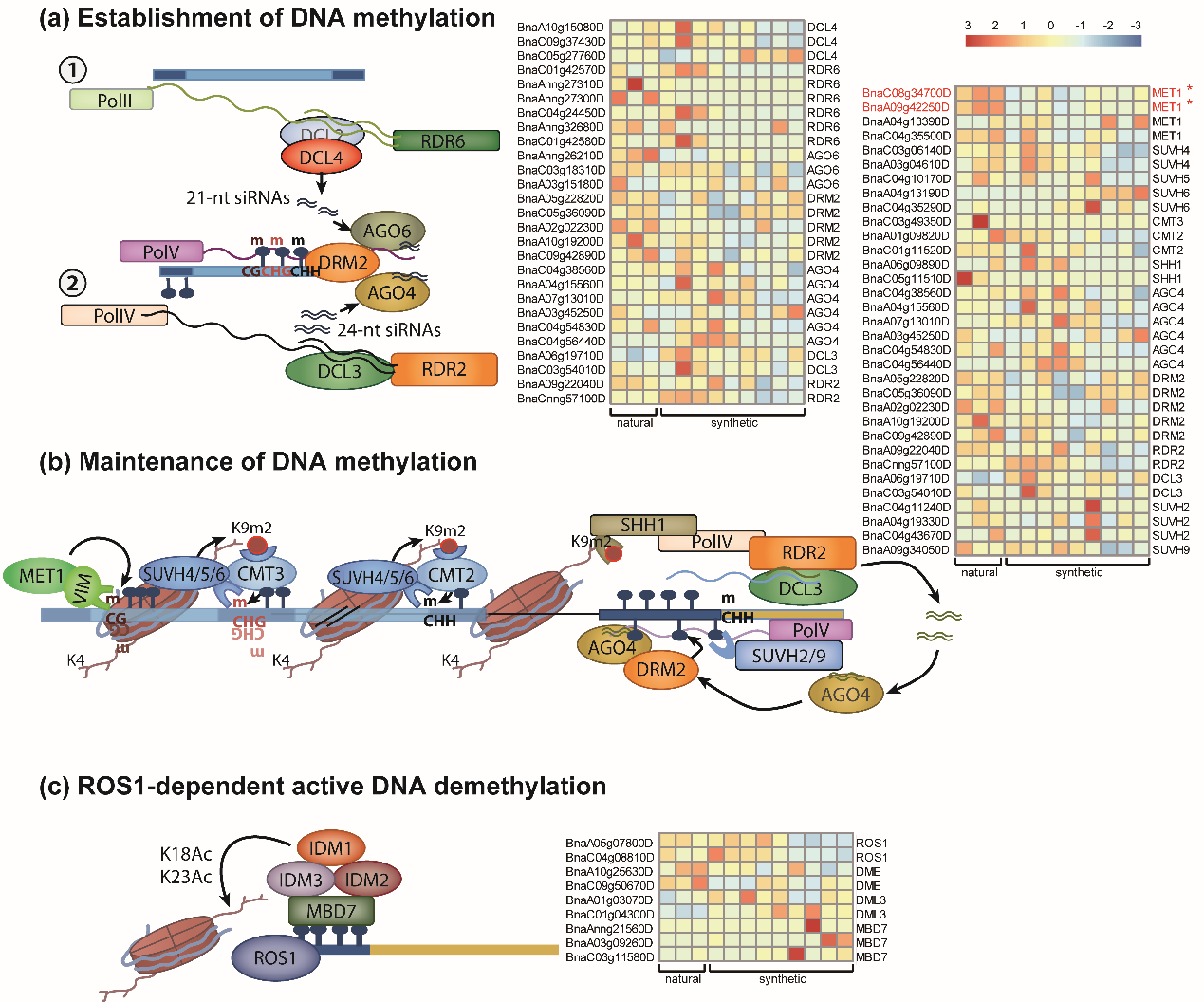


**Fig. S1** Expression of genes involved in the establishment, maintenance, and removal of DNA methylation. Red font indicates that the genes were differentially expressed between natural and synthetic rapeseeds. The model is from Deleris, et al [^1^](#_ENREF_1).


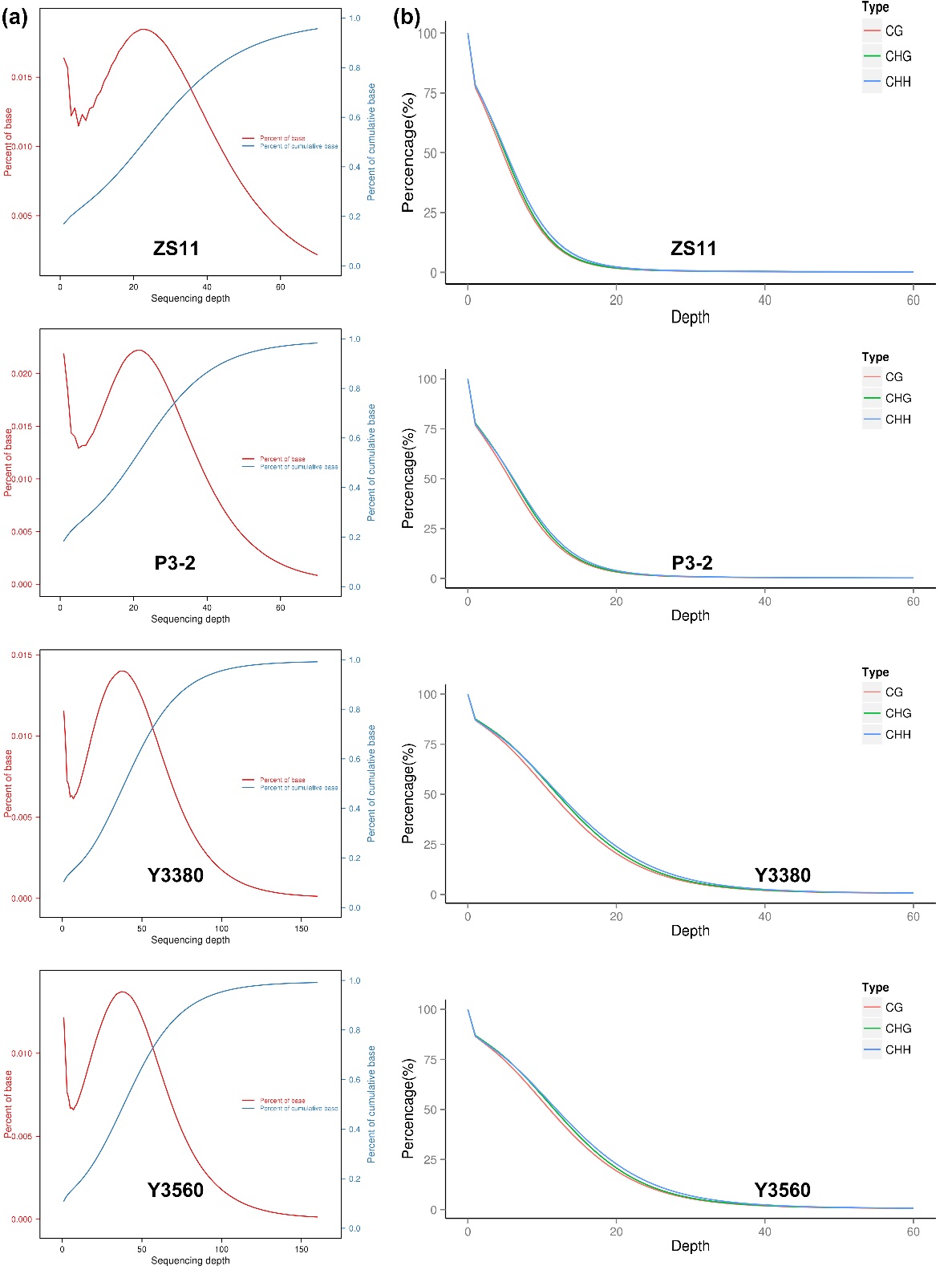


**Fig. S2** Sequencing depth of WGBS. (**a**) Sequence depth distribution. The abscissa is the sequencing depth, the left ordinate is the percentage of bases corresponding to this depth (blue curve), and the right ordinate is the percentage of bases below this depth (red curve). (**b**) Cumulative distribution of cytosine base coverage. The abscissa is the coverage depth of cytosine sites, and the ordinate is the percentage of cytosine sites greater than or equal to the corresponding coverage depth to the total cytosine sites.


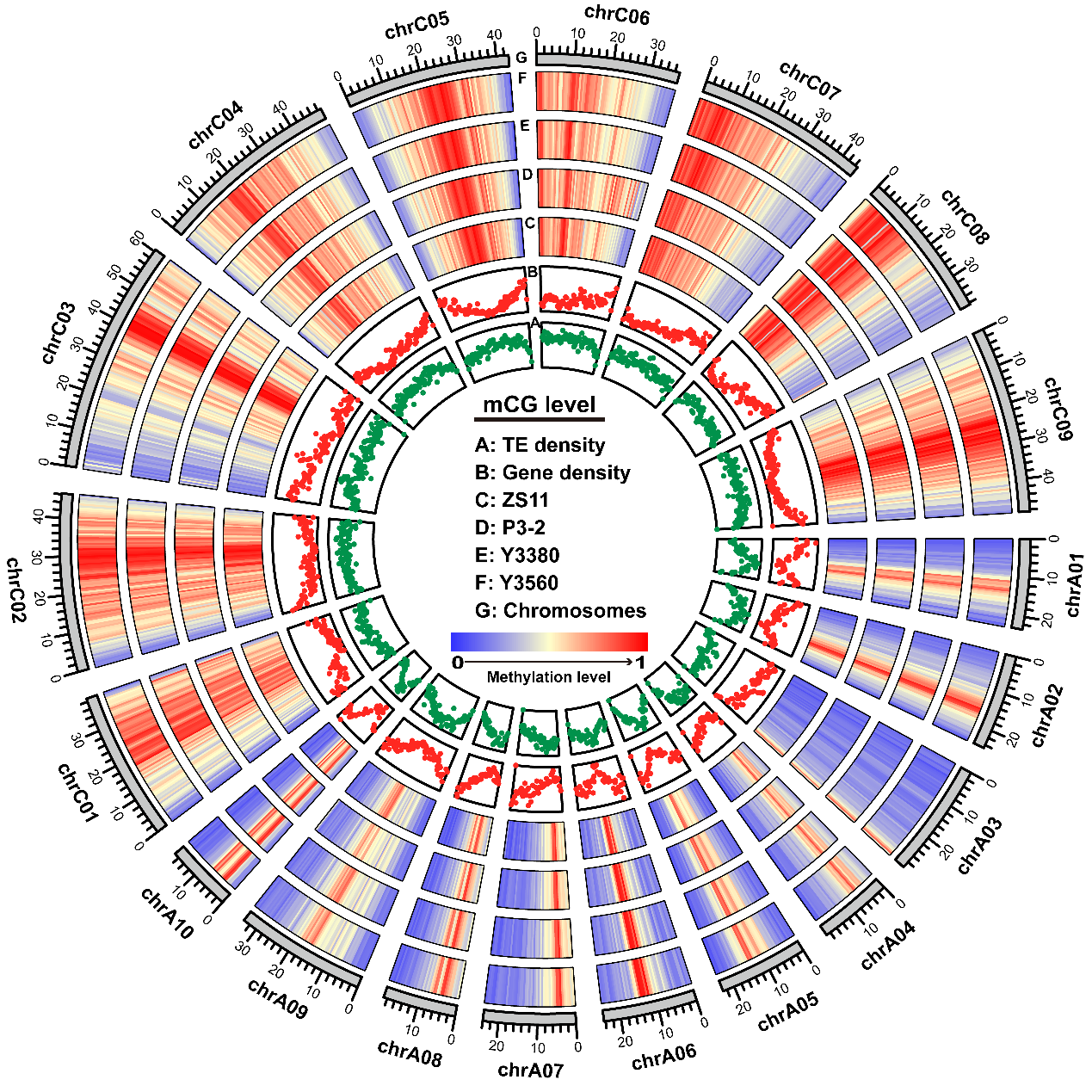


**Fig. S3** Circos plot of the mCG level. Track order from inside to outside: the density of TEs; density of genes; mCG level of ZS11; mCG level of P3-2; mCG level of Y3380; mCG level of Y3560; and chromosomes.


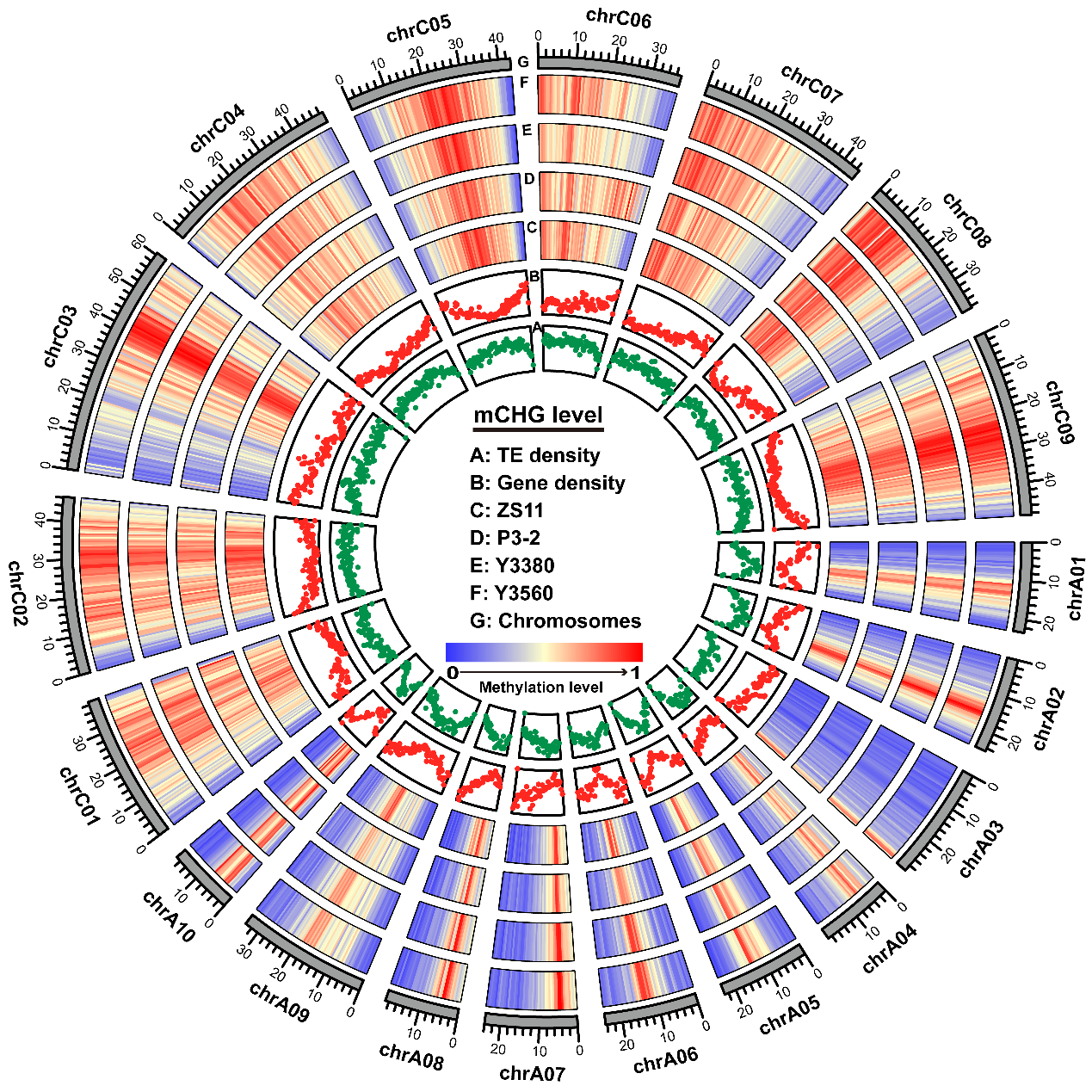


**Fig. S4** Circos plot of the mCHG level. Track order from inside to outside: the density of TEs; density of genes; mCHG level of ZS11; mCHG level of P3-2; mCHG level of Y3380; mCHG level of Y3560; and chromosomes.


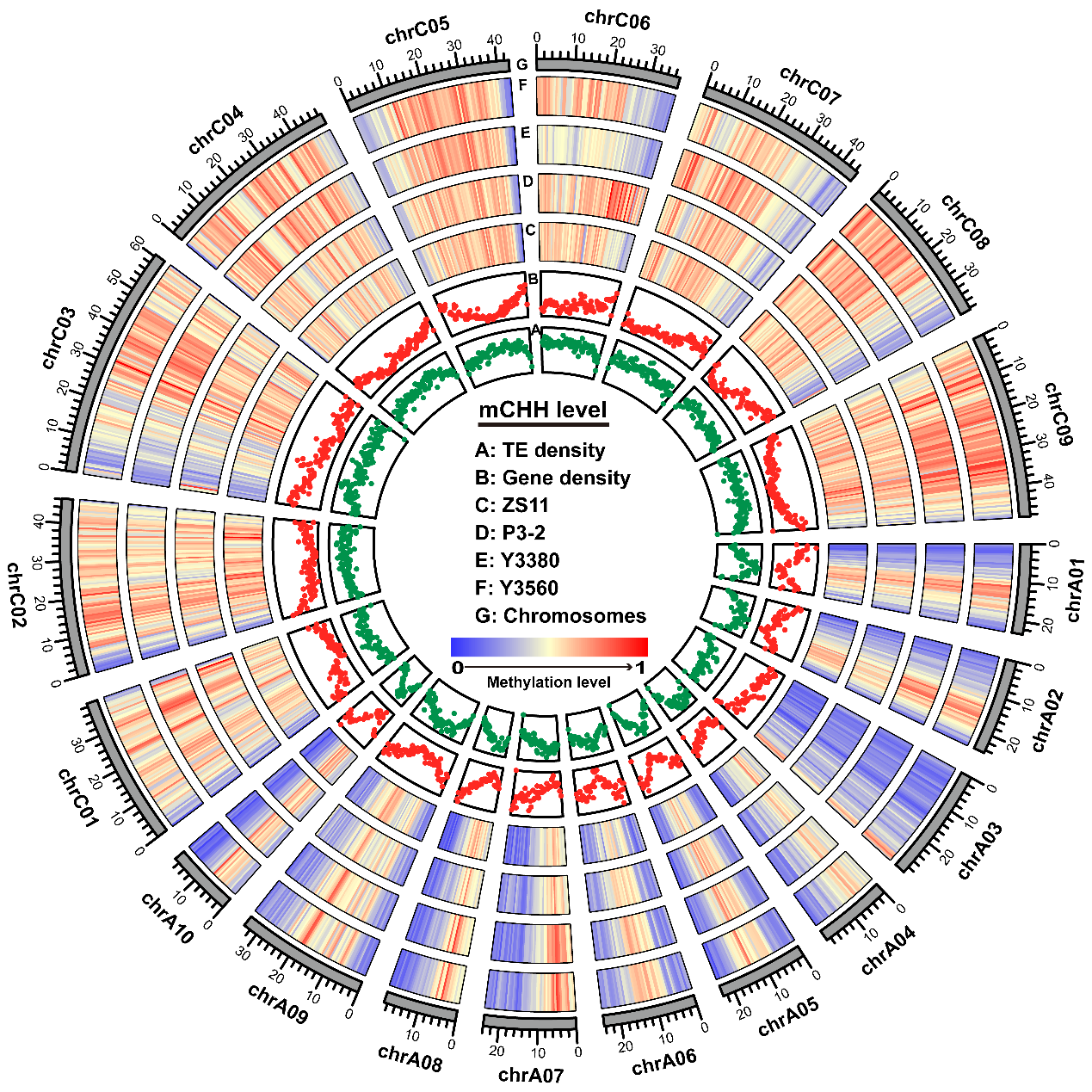


**Fig. S5** Circos plot of the mCHH level. Track order from inside to outside: the density of TEs; density of genes; mCHH level of ZS11; mCHH level of P3-2; mCHH level of Y3380; mCHH level of Y3560; and chromosomes.


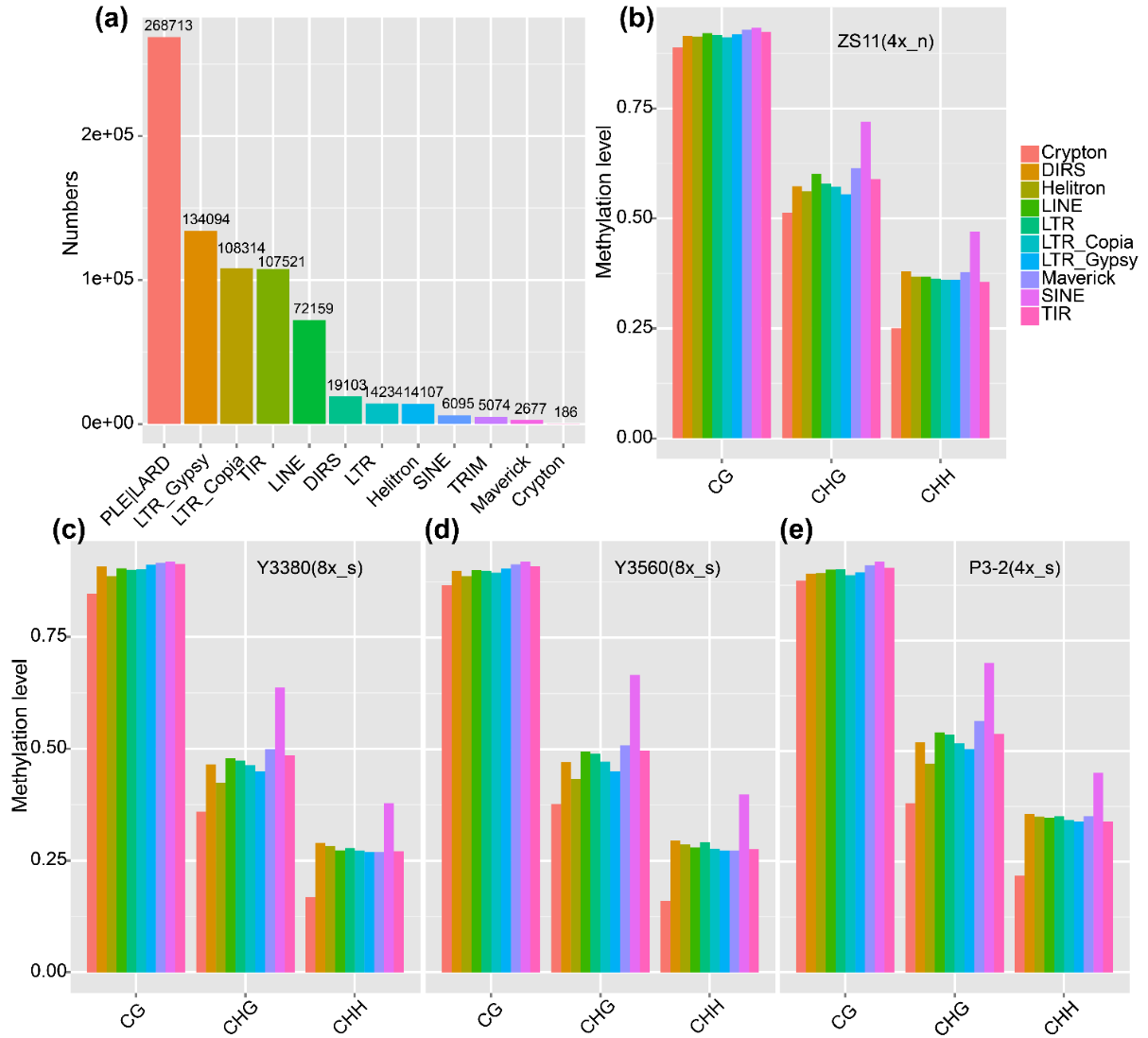


**Fig. S6** Methylation patterns of TEs in the *B. napus* genome. (**a**) Numbers of different types of TEs in the *B. napus* genome. (**b**) Methylation levels of different types of TEs in the natural *B. napus* ZS11. (**c**) Methylation levels of different types of TEs in the synthetic octoploid *B. napus* 3380. (**d**) Methylation levels of different types of TEs in the synthetic octoploid *B. napus* 3560. (**e**) Methylation levels of different types of TEs in the synthetic *B. napus* P3-2.


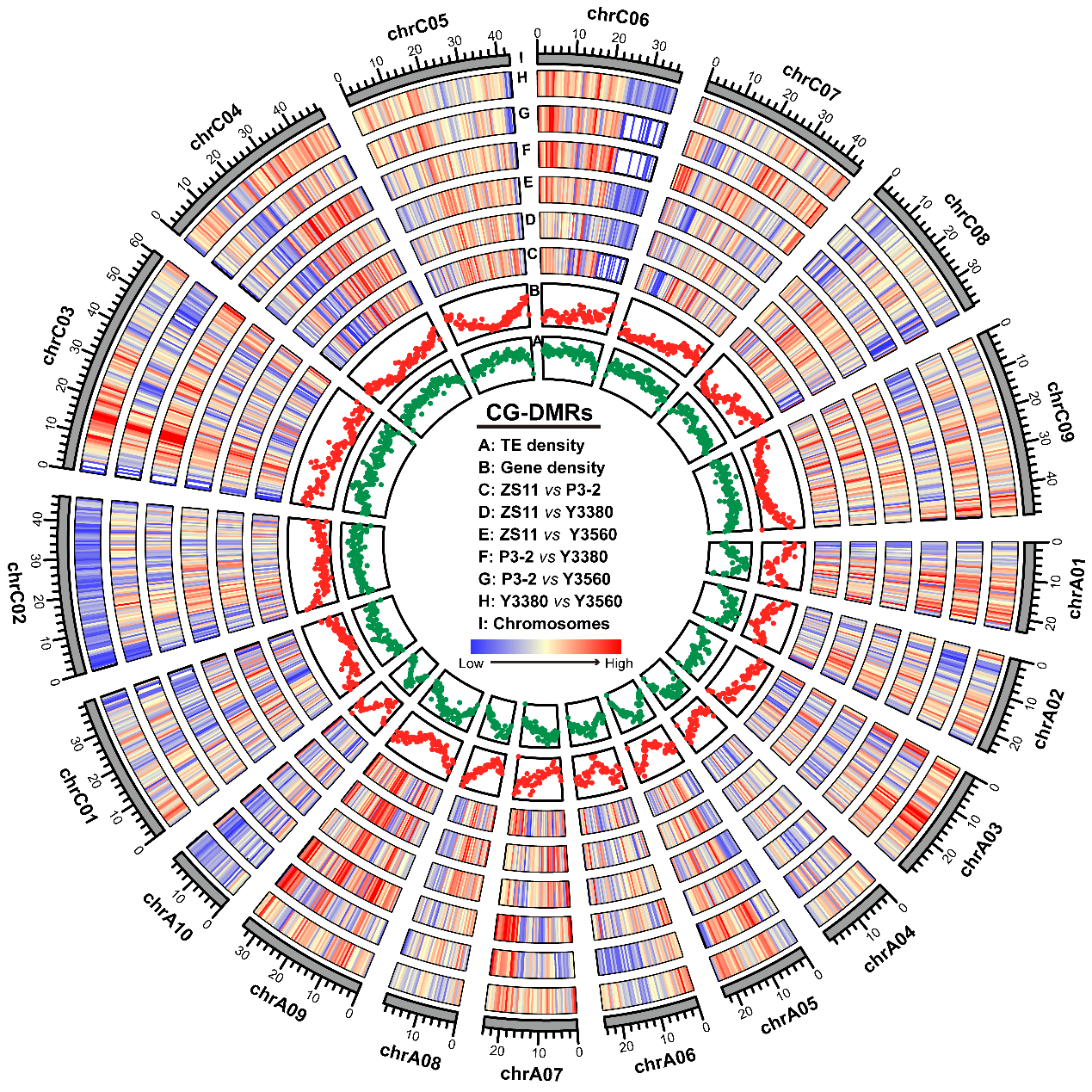


**Fig. S7** Genome-wide distribution of CG-DMRs. Track order from inside to outside: the density of TEs; density of genes; density of CG-DMRs from ZS11 *vs* P3-2; density of CG-DMRs from ZS11 *vs* Y3380; density of CG-DMRs from ZS11 *vs* Y3560; density of CG-DMRs from P3-2 *vs* Y3380; density of CG-DMRs from P3-2 *vs* Y3560; density of CG-DMRs from Y3380 *vs* Y3560; and chromosomes.


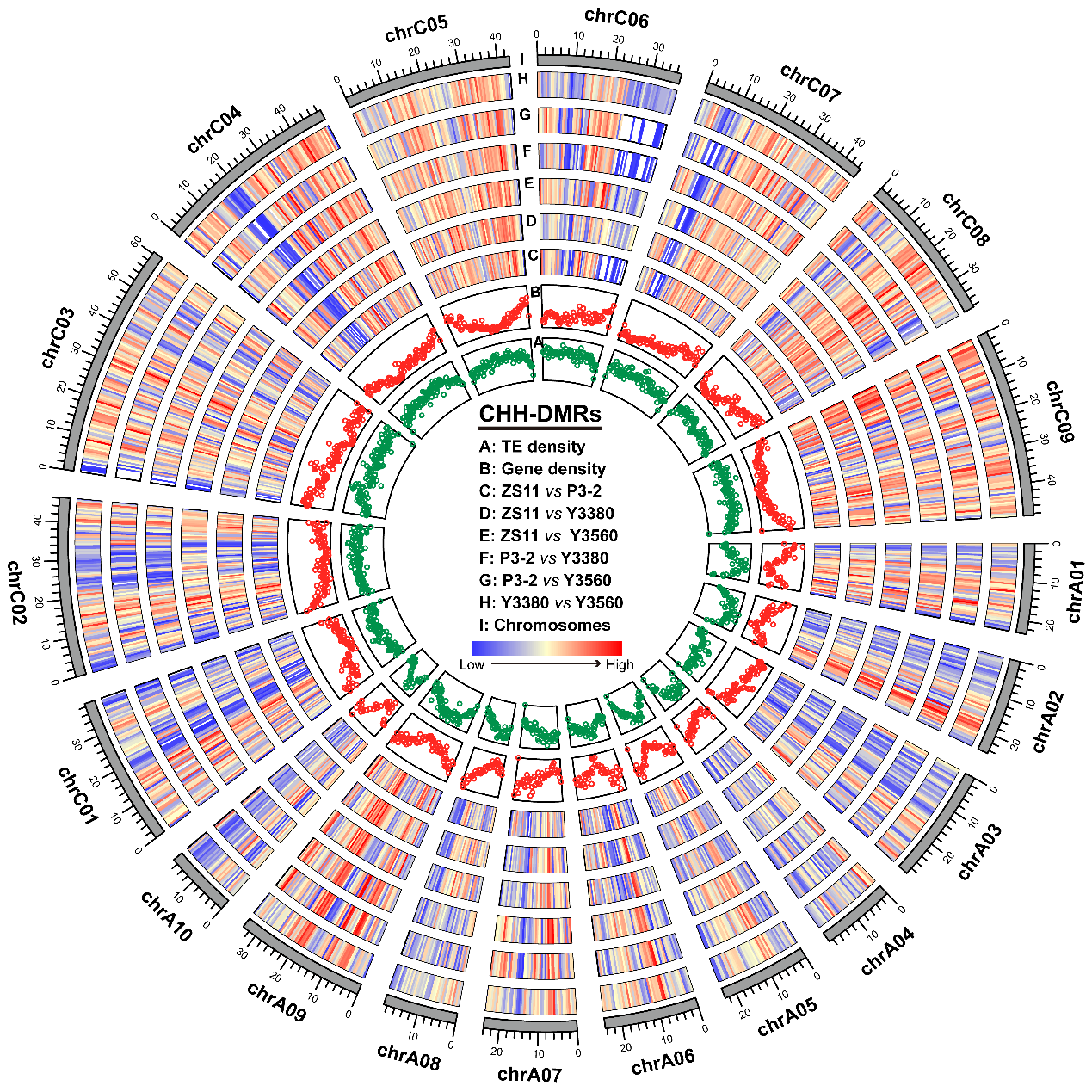


**Fig. S8** Genome-wide distribution of CHH-DMRs. Track order from inside to outside: the density of TEs; density of genes; density of CHH-DMRs from ZS11 *vs* P3-2; density of CHH-DMRs from ZS11 *vs* Y3380; density of CHH-DMRs from ZS11 *vs* Y3560; density of CHH-DMRs from P3-2 *vs* Y3380; density of CHH-DMRs from P3-2 *vs* Y3560; density of CHH-DMRs from Y3380 *vs* Y3560; and chromosomes.


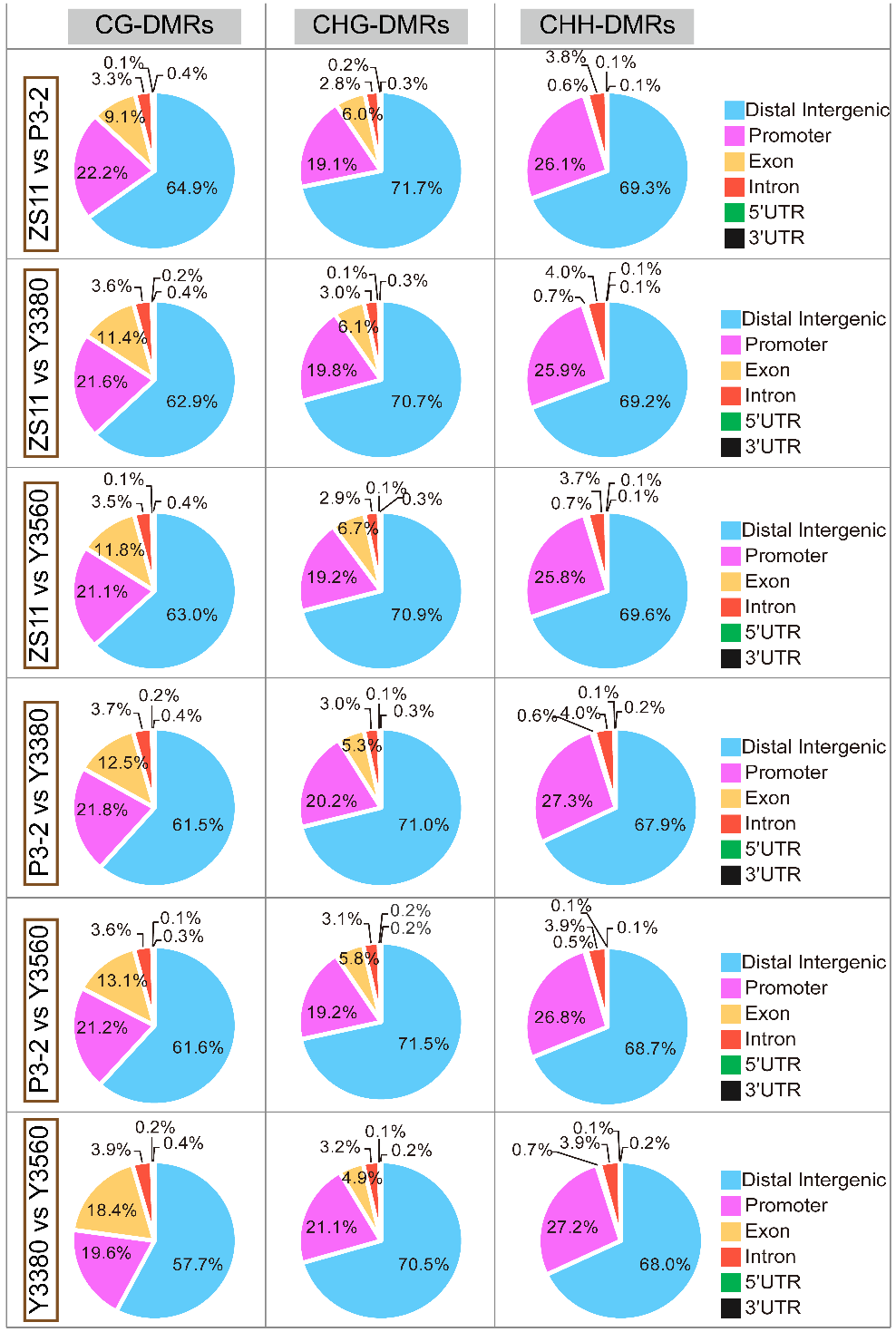


**Fig. S9** Genome-wide distributions of DMRs in the three sequence contexts. Horizontally from left to right: CG-DMRs, CHG-DMRs and CHH-DMRs. Vertically from top to bottom: pairwise comparisons of ZS11 *vs* P3-2, ZS11 *vs* Y3380, ZS11 *vs* Y3560, P3-2 *vs* Y3380, P3-2 *vs* Y3560, and Y3380 *vs* Y3560.


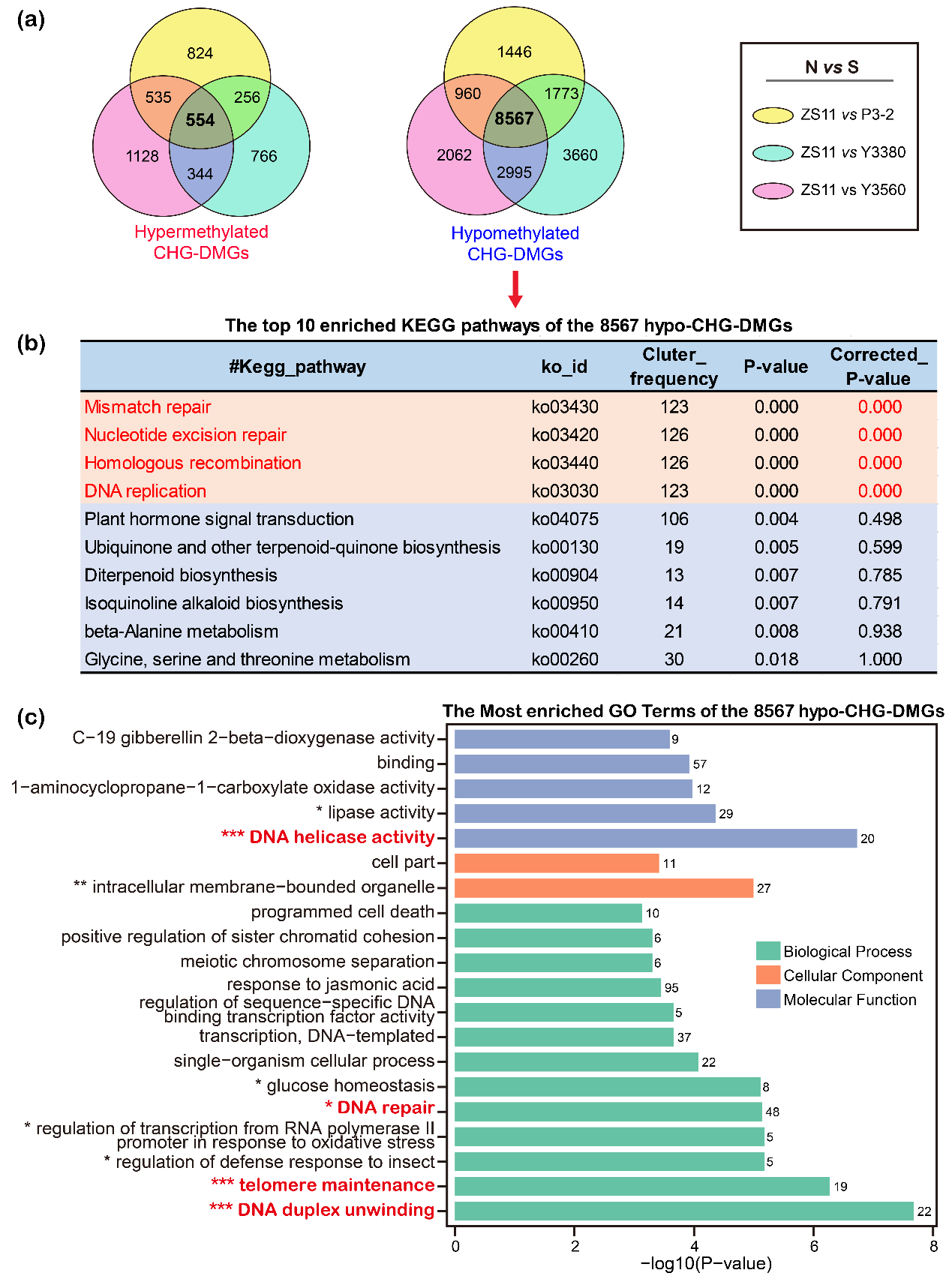


**Fig. S10** Common CHG-DMGs among the three N *vs* S comparisons (**a**) and the enrichment analysis (**b**-**c**) of the common hypomethylated CHG-DMGs (hypo-CHG-DMGs). *, corrected p-value < 0.05; **, corrected p-value < 0.01; **, corrected p-value < 0.001. This figure has been added to the revised Supplemental Figures.


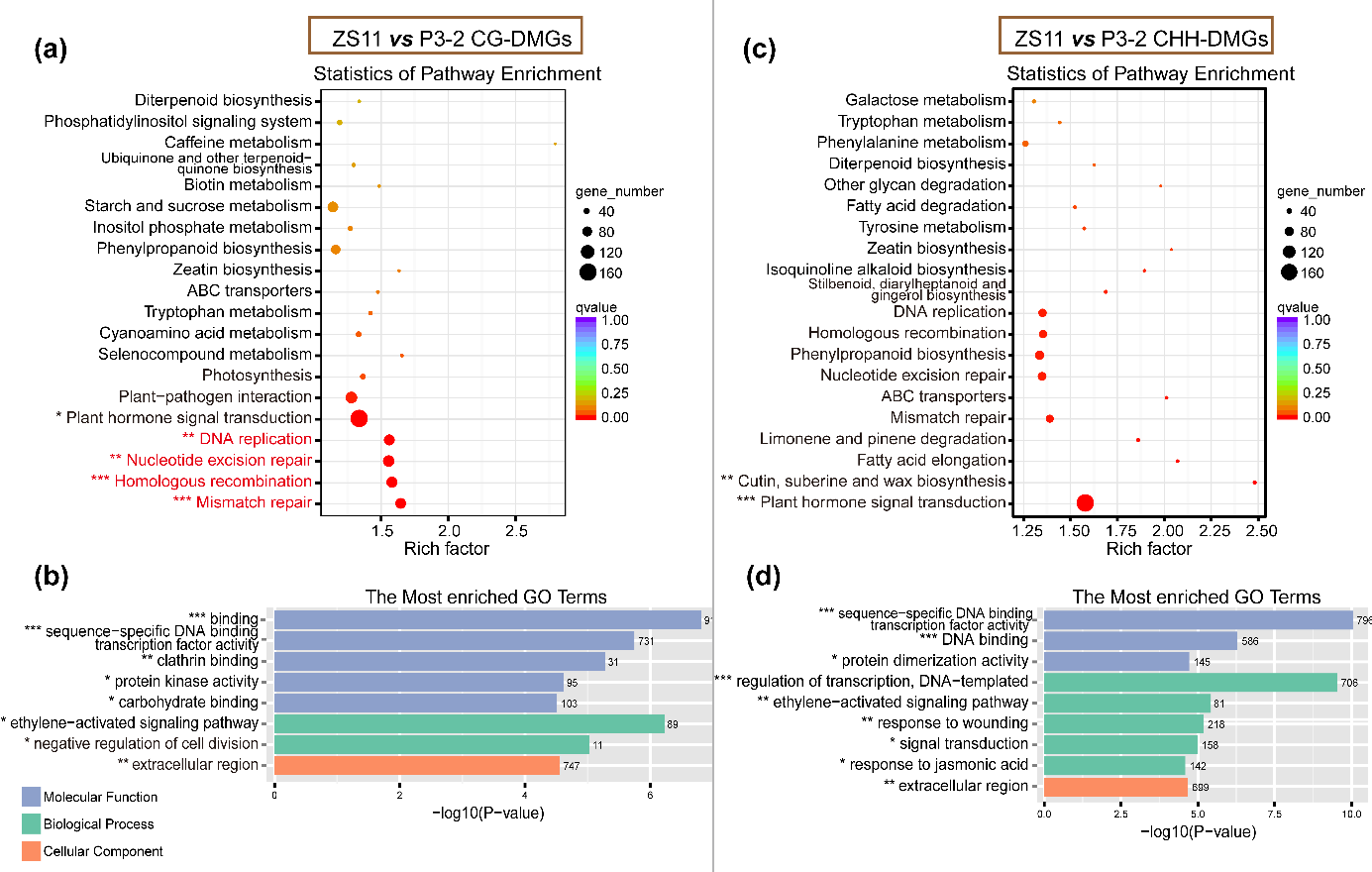


**Fig. S11** Functional enrichment analysis of CG-DMGs and CHH-DMGs between ZS11 and P3-2. (**a**) KEGG enrichment of CG-DMGs. (**b**) GO enrichment of CG-DMGs. (**c**) KEGG enrichment of CHH-DMGs. (**d**) GO enrichment of CHH-DMGs. Only the significant GO terms (corrected *P*-value <0.05) are listed. ***: corrected *P*-value < 0.001; **: corrected *P*-value < 0.01; *: corrected *P*-value < 0.05.


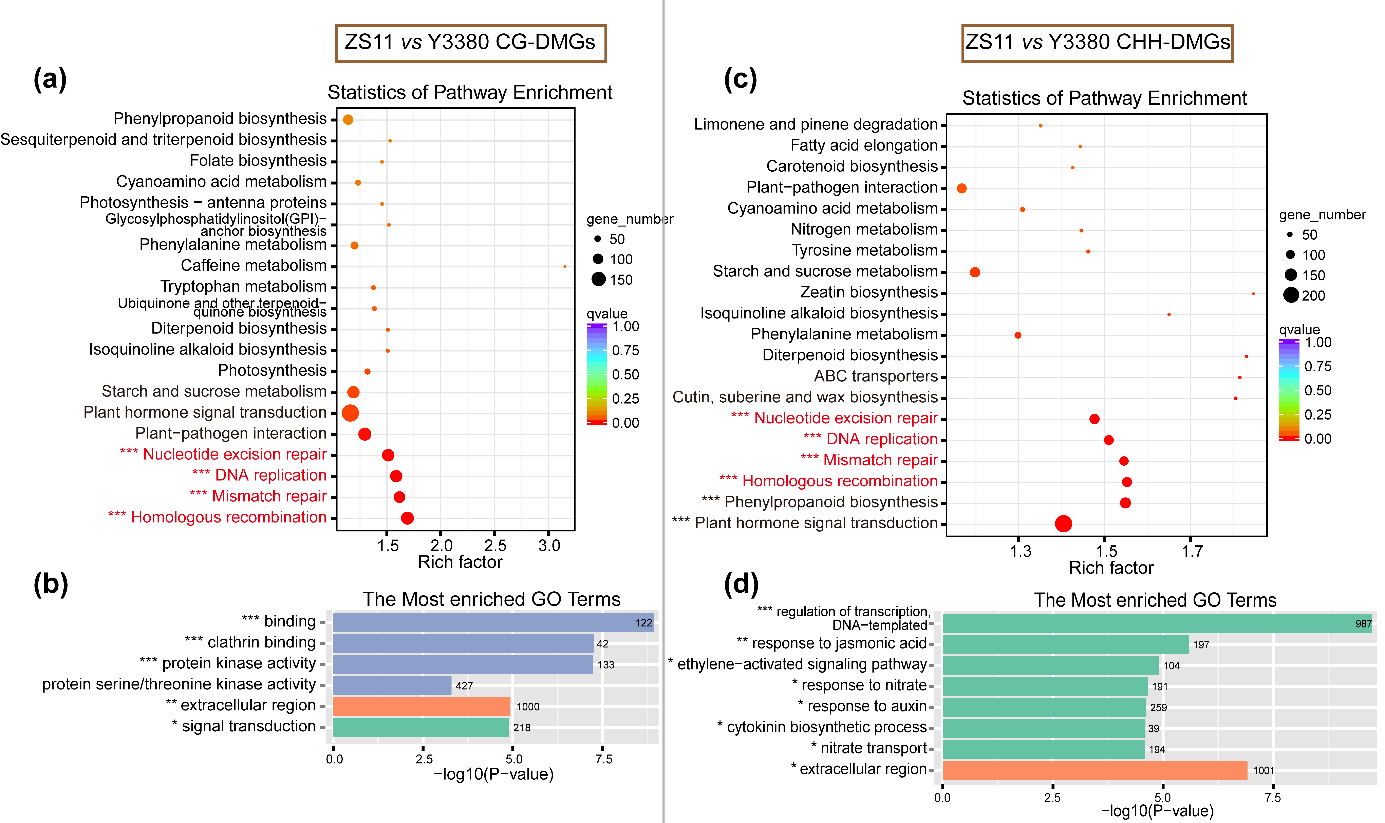


**Fig. S12** Functional enrichment analysis of CG-DMGs and CHH-DMGs between ZS11 and Y3380. (**a**) KEGG enrichment of CG-DMGs. (**b**) GO enrichment of CG-DMGs. (**c**) KEGG enrichment of CHH-DMGs. (**d**) GO enrichment of CHH-DMGs. Only the significant GO terms (corrected P-value <0.05) are listed. ***: corrected P-value < 0.001; **: corrected P-value < 0.01; *: corrected P-value < 0.05.


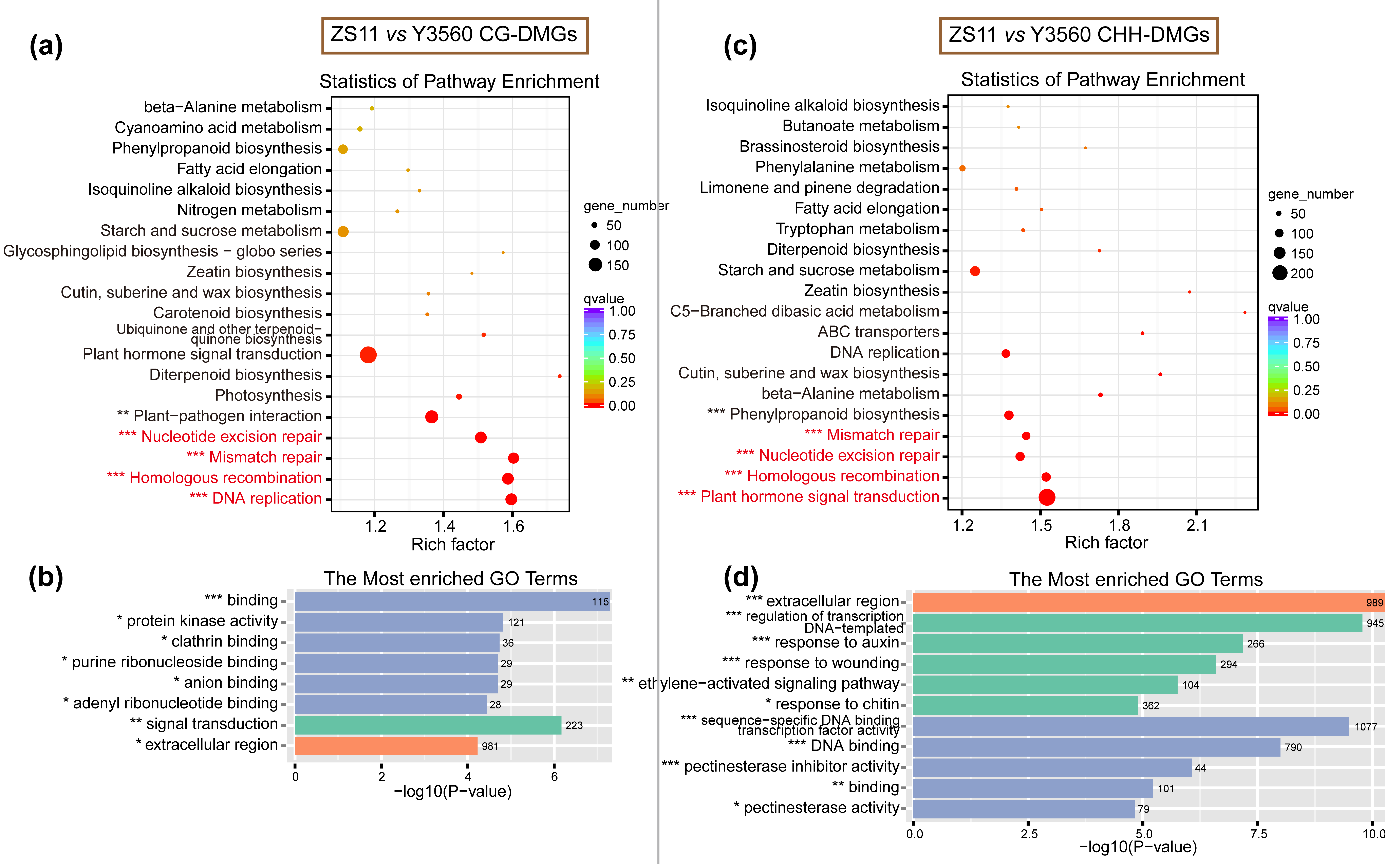


**Fig. S13** Functional enrichment analysis of CG-DMGs and CHH-DMGs between ZS11 and Y3560. (**a**) KEGG enrichment of CG-DMGs. (**b**) GO enrichment of CG-DMGs. (**c**) KEGG enrichment of CHH-DMGs. (**d**) GO enrichment of CHH-DMGs. Only the significant GO terms (corrected P-value <0.05) are listed. ***: corrected P-value < 0.001; **: corrected P-value < 0.01; *: corrected P-value < 0.05.


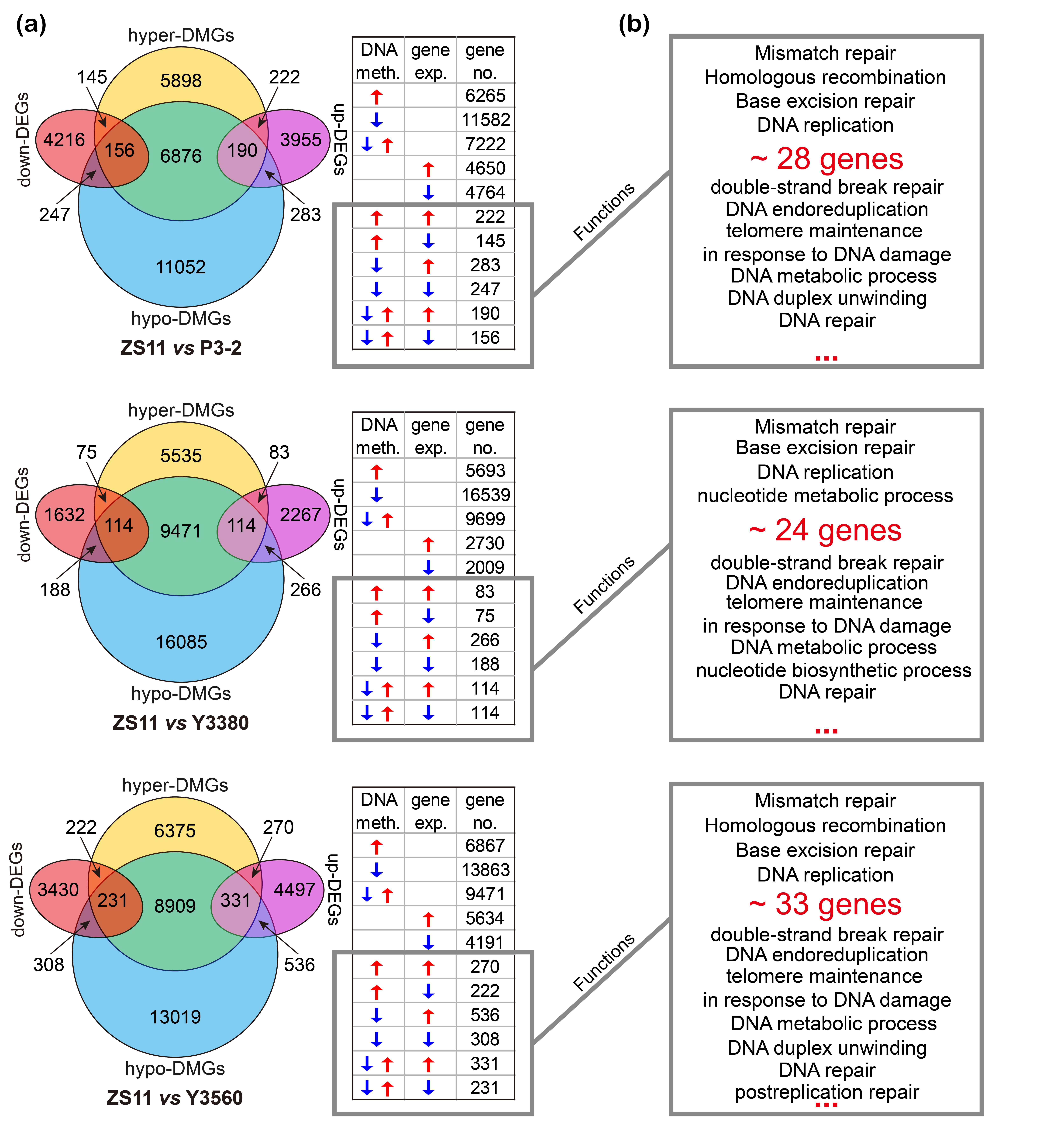


**Fig. S14** Relationship between DMGs and DEGs. (**a**) Venn diagram of DEGs and DMGs between natural and synthetic rapeseeds. **↑↓** indicates that the DMGs overlapped with both hypo- and hyper-DMRs. (**b**) Part of the functional enrichment of the genes that were both differentially methylated and differentially expressed between samples. The red numbers indicate the number of genes enriched in pathways and GO terms related to DNA repair and metabolism.


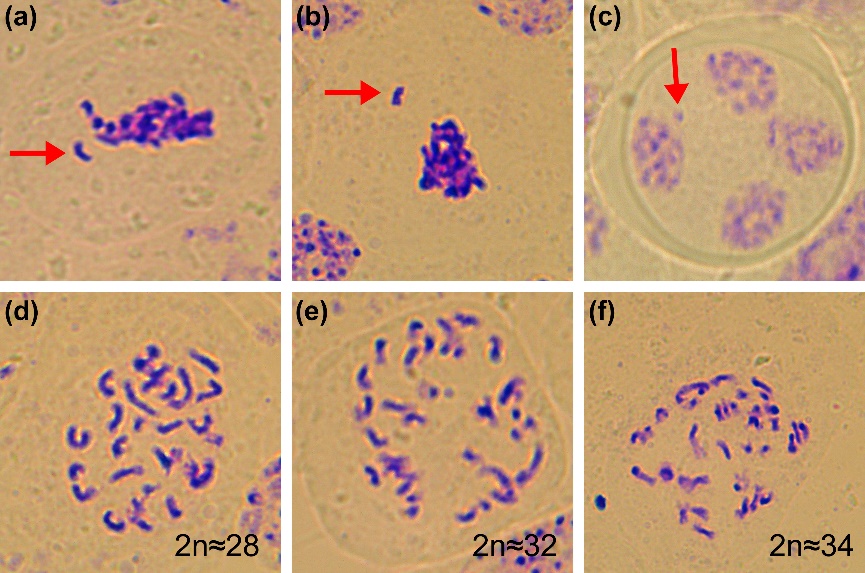


**Fig.** **S15** Synthetic *B. napus* P3-2 (2*n* = 38) with abnormal meiosis (**a-c**) and its self-pollinated progenies containing aneuploid chromosomes (**d-f**).

**References**

1. Deleris, A., Halter, T. & Navarro, L. DNA methylation and demethylation in plant immunity*. Annu. Rev. Phytopatho*l**.** 54, 579-603 (2016).
